# Control of perovskite film crystallization and growth direction to target homogeneous monolithic structures

**DOI:** 10.1038/s41467-022-34332-3

**Published:** 2022-11-04

**Authors:** Daming Zheng, Florian Raffin, Polina Volovitch, Thierry Pauporté

**Affiliations:** 1 Nanophotonics Research Center, Shenzhen Key Laboratory of Micro-Scale Optical Information Technology & Institute of Microscale Optoelectronics, Shenzhen University, 518060 Shenzhen, China; 2 Chimie ParisTech, PSL Research University, CNRS, Institut de Recherche de Chimie Paris (IRCP), UMR8247, 11 rue P. et M. Curie, F-75005 Paris, France

**Keywords:** Energy, Devices for energy harvesting

## Abstract

Getting performant organo-metal halide perovskite films for various application remains challenging. Here, we show the behavior of solvent and perovskite elements for four different perovskites families and nine different initial precursor solution systems in the case of the most popular preparation process which includes an anti-solvent dripping-assisted spin coating of a precursor solution and a subsequent thermal annealing. We show how the initial solution composition affects, first, the film formed by spin coating and anti-solvent dripping and, second, the processes occurring upon thermal annealing, including crystal domain evolution and the grain growth mechanism. We propose a universal typology which distinguishes three types for the growth direction of perovskite crystals: downward (Type I), upward (Type II) and lateral (Type III). The latter results in large, monolithic grains and we show that this mode must be targeted for the preparation of efficient perovskite light absorber thin films of solar cells.

## Abstract (web summary)

Organo-metal halide perovskites are promising semiconductor materials for active layers applied to opto-electronics, e.g. photovoltaic cells. Here authors show how the initial solution composition affects the formation of perovskite thin films, and propose a typology to distinguish the growth direction of perovskite crystals.

## Introduction

Organo-metal halide perovskites (MHPs), with general formula ABX_3_ (with A being a monovalent cation, B being Pb or Sn and X being an halogen ion), have emerged among the most promising semiconductor materials for the preparation of the active layers applied to opto-electronics, especially photovoltaic solar cells^1–6^. They combine a facile preparation from perovskite precursor solutions (PPSs), manufacturing at mild temperature, a high tunability of their optical and electronic properties, up-scaling potential and low production costs^7–12^. Fast progress in MHP research has been possible by a great global effort of the scientific community which has notably allowed a rapid improvement of the final films properties by playing on their final composition, on the perovskite precursor solution (PPS) composition^13–16^, interfacial engineering^8,14^, as well as on the processing techniques employed to obtain the final product^17,18^.

For photovoltaic (PV) applications, the quality of the final layer is a key parameter for both high performance and device robustness and stability. The final targeted film properties are a good coverage of the substrate, with no pinholes nor voids. The grains composing the films must be large, well-crystallized and with a monolithic structure. The defects must be passivated. In the monolithic structure, each grain is contacted by the adjacent layers which renders the transfer of the photogenerated charges fast and efficient. The growth and final properties are controlled, not only by the deposition technique and annealing parameters, but also by the components introduced in the PPS which are divided into those which will enter into the final composition of the layer and those that will help the synthesis but will be eliminated, especially upon the thermal annealing step. They are dissolved in high-boiling point polar solvents, typically N,N’-dimethylformamide (DMF), dimethyl sulfoxide (DMSO), γ-butyrolactone (GBL), N-methyl-2-pyrrolidone (NMP), and others^19^. They are often mixed together or with a low-boiling point solvent for a better control of the precursors solubilization and elimination upon the film formation process. At present, the record solar cells have been fabricated from MHP films deposited by spin coating^20^. To accurately control the state of the layer that will be subsequently annealed, the most popular way consists in dripping an anti-solvent such as chlorobenzene, diethyl ether, or toluene upon spinning^21–23^. This step must be performed at a well-defined time which depends on the PPS composition and temperature. This technique is usually called one-step technique^24,25^ in contrast with those where a precursor film, such as a PbI_2_ film, is reacted with a monovalent cation solution or vapor to form the perovskite^17,26,27^. Despite the huge experimental knowledge and work accumulated on how performant MHP films for PV can be produced, the mechanism of film formation, especially upon spin coating and thermal annealing has not been fully unveiled yet. How, starting from the wet film, the solvent is eliminated, and the crystal growth is controlled to get the monolithic structure upon thermal annealing, remains to be understood. For this, one must know how perovskite grains are formed from the wet layer that results from the spin coating and anti-solvent dripping. Techniques must be implemented to follow the fate of solvent molecules and various ions and their profile across the layer’s thickness evolution.

If several analytical techniques are available to detect the composition of organic-inorganic perovskite films, only a few of them allow an accurate depth profiling of the films and of the whole devices. In the present work, the glow discharge-optical emission spectroscopy (GD-OES) technique has been especially employed^28^, combined with x-ray diffraction (XRD) and scanning electron microscopy (SEM). GD-OES is now well-established for elemental depth profiling of multilayered systems with layers thickness ranging from several nm to hundreds µm^29^. The analytical capacities of GD-OES for the element detection can be as low as several ppm and its analytical capacities for depth profiling of thin layers are comparable with high vacuum surface analytical techniques such as Auger electron spectroscopy (AES) or X-ray photoelectron spectroscopy (XPS)^30–32^. However, the depth profiling by the latter techniques is limited to only nanometric depths and to a reduced surface area of several hundred µm^2^, they are thus insufficient to reflect the overall situation of perovskite films and perovskite solar cells (PSCs) and they are prone to errors. Even for Hard X-ray Photoelectron Spectroscopy (HAXPES) technology which provides chemical information several times deeper than conventional XPS, its depth of analysis is still limited, and a full perovskite film employed in PSC cannot be analyzed.^33^. On the other hand, the analyzed zone of GD-OES is determined by the anode size, which can be several tens of mm^2^ ^31^. This technique appears to be an interesting tool for depth profiling of MHP solar materials. The fact that the sputtering direction is perpendicular to the surface decreases the shadowing effect during sputtering. This makes possible to apply GD-OES to the analysis of the films formed on rough substrates^34^. Quantitative analysis of thin films by GD-OES can be tricky^35,36^; however, qualitative depth profiles analysis is also very informative and has been previously successfully applied in stability evaluation of perovskite materials^37–39^. However, to our knowledge, the unique GD-OES capabilities, notably the fast profile recording (several tens of seconds for a solar cell perovskite layer) has poorly been applied to the study of the perovskite film preparation and to the film growth analysis^16^.

In the present paper, we investigate the solvent and perovskite elements location in the films and their profile evolution upon the full film formation process. To get a view as large as possible and draw sound conclusions, we have investigated four different perovskite families, namely MAPbI_3_, FAPbI_3_/ FA_1-x_MA_x_PbI_3_, Cs_0.1_FA_0.9_PbI_3_, and Rb_0.05_Cs_0.05_FA_0.9_PbI_3_ (with MA for methylammonium and FA for formamidinium) and nine different PPS compositions, including or not halides and metal nanoparticle additives. They have allowed us to state two universal laws concerning the films produced by spin coating and subsequent thermal annealing. First, when the perovskite film is prepared by spin coating, the solvent accumulates in the upper part of the layer: the upper layer is richer in solvent than the lower one. On the other hand, Pb of perovskite is concentrated in the layer lower part. This surface solvent excess is eliminated upon the first seconds of annealing. Second, we have observed and analyzed three types of growth direction of perovskite crystals upon thermal annealing: upward, downward, and lateral. Among them, PSCs with high efficiency are always a consequence of the lateral growth. Furthermore, the growth direction is the results of the microstructural and compositional properties of the film that results from the spin coating/anti-solvent dripping step. We have been able to change the growth direction of perovskite crystals in the film by using accurate additives, especially halide salts or gold nanoparticles.

## Results

### Investigated MHP films and solar cells

**Fig. 1:**
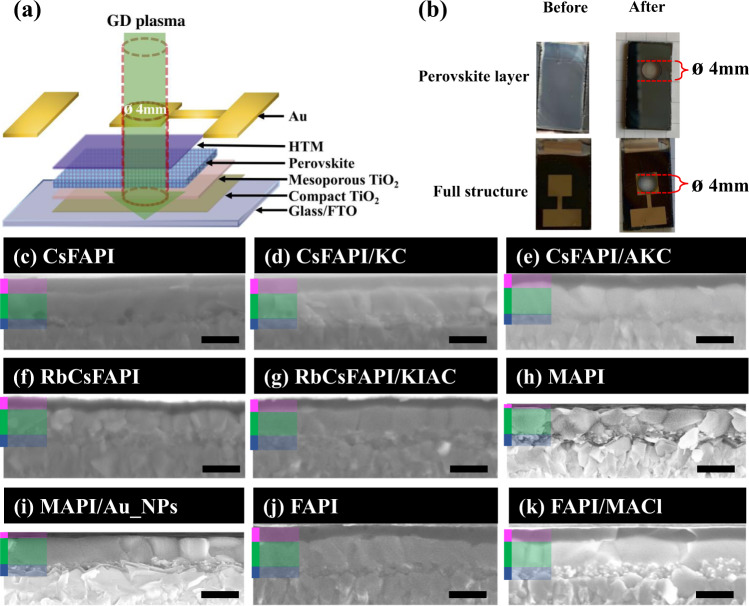
Cross-sectional investigation of the layer stacks. **a** Investigated solar cells architecture with the scale of the GD plasma beam passing through the perovskite solar cell (the detection diameter is 4 mm, HTM is the hole transporting material). **b** Perovskite film and full structure before and after their investigation by GD-OES. **c**–**k** Cross-sectional SEM views of solar cells based on **c** pristine CsFAPI, **d** CsFAPI with KCl additive, and **e** with KCl and NH_4_Cl additives; **f** Pristine RbCsFAPI and (**g**) RbCsFAPI with KI and NH_4_Cl additives; **h** MAPI and **i** MAPI with gold nanoparticles additive; **j** Pristine FAPI and **k** FAPI with MACl additive. (green: Perovskite; Blue: mesoporous TiO_2_ and perovskite; Magenta: Spiro-OMeTAD. Scale bar: 400 nm).

The perovskite films investigated in the present work had various compositions and their deposition protocols have been optimized in our group in the past years. The solar cell structure is shown in Fig. 1a. The MHP film was contacted by an n-type mesoporous TiO_2_ film on one side and by a p-type Spiro-OMeTAD layer on the other side. All the films were obtained by spin coating with the dropping of chlorobenzene anti-solvent upon spinning. The films were subsequently annealed at an optimized temperature.

The investigated perovskites are divided into four families and nine samples. CsFAPI are iodide perovskites which contain Cs and formamidinium and of Cs_0.1_FA_0.9_PbI_3_ general chemical formula. RbCsFAPI contains in addition rubidium cation (Rb_0.05_Cs_0.1_FA_0.85_PbI_3_). MAPI is methylammonium lead iodide (MAPbI_3_) and FAPI is formamidinium lead iodide (FAPbI_3_). Additives have been employed to assist the perovskite formation and improve the performance. The tested halide additives include KCl (noted KC), KI, NH_4_Cl (ammonium chloride, noted AC) and MACl. AKC is the notation for the layer in which both KCl and NH_4_Cl additives were mixed. With MACl, the obtained compound was FA_1-x_ MA_x_PbI_3_ since a small fraction of MA^+^ is incorporated into the lattice while most of MA^+^ is eliminated upon thermal annealing^40,41^. Gold nanoparticles (Au_NPs) with a mean diameter of 14 nm were also investigated as additives to assist the preparation of MAPbI_3_. The details of perovskite film and solar cell preparations are given in the Supplementary Information.

**Fig. 2:**
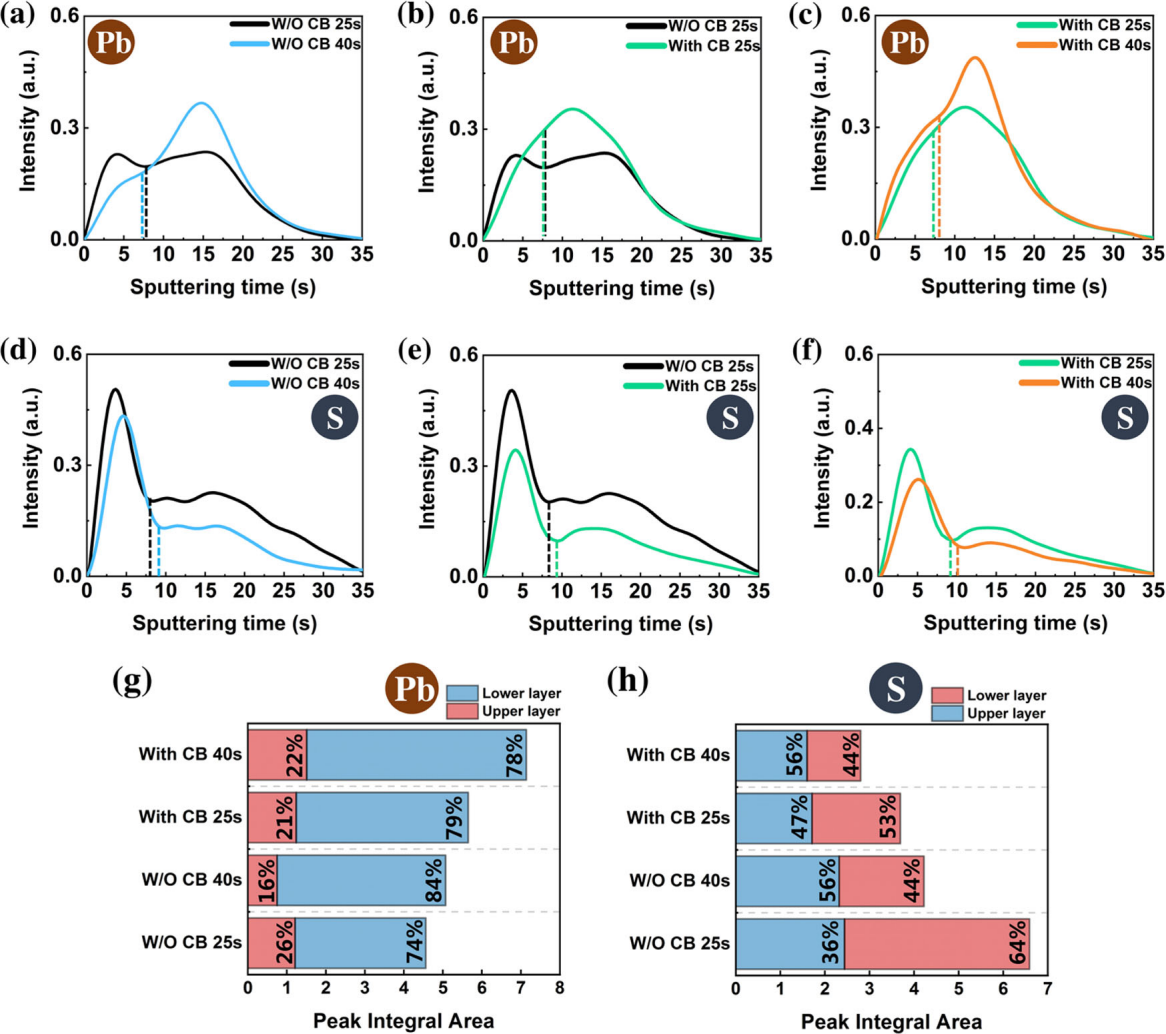
GD-OES investigation of spin coating and dripping processes. Evolution of GD-OES elemental depth profiles. **a**–**c** lead (Pb) and **d**–**f** sulfur (S) in the CsFAPI/AKC perovskite precursor layer upon spin coating (sputtering erosion from top to bottom). The interface between the upper and lower layers is indicated by a dashed line on each profile. Absolute values of the area under the curve delimited by each layer are shown for Pb (**g**) and S (**h**) and the numbers indicate the relative contributions of the layers to the total area under the curves. Source data are provided as a Source Data file.

The list of the investigated samples with their names, compositions and additives is given in Table 1. This table also provides the *J-V* curves characteristics for the champion solar cells and their hysteresis index. As stated in our previous work, the hysteresis between the reverse and forward *J-V* scans is suppressed when a potassium halide salt (KI or KCl) is added to the PPS^16^. K^+^ is not eliminated upon annealing, it passivates the defects at the origin of the iodide ion mobility^16,31,42,43^. Moreover, in our Ref. 31, we have clearly established the correlation between the I mobility blocking and the *J-V* curve hysteresis suppression. Table 1 reports the PSCs prepared with untreated MHP films, while Supplementary Table 1 shows that the MHP film treatment with a n-propylammonium iodide (PAI) solution or a 2-phenylethylammonium iodide (PEAI) solution^14^ improves the final PCE of the devices and that our best systems presented an efficiency above 22%. The *J-V* curves and the statistics of the photovoltaic parameters of the solar cells are presented in Supplementary Fig. 1 and Fig. 2, respectively.

**Table 1:** List of studied samples and solar cells

Sample	Perovskite	Additive	Scan direction	*V*_*oc*_ [V]	*J*_*sc*_ [mA. cm^−2^]	*FF* [%]	PCE [%]	*HI*^a^ [%]
CsFAPI	Cs_0.1_FA_0.9_PbI_3_	No	Reverse	0.943	23.91	72.13	16.26	26
			Forward	0.846	23.83	59.36	11.96	
CsFAPI/KC	K_0.09_Cs_0.1_FA_0.81_PbI_3_	KCl	Reverse	0.994	25.19	76.38	19.12	1
			Forward	0.995	25.16	76.03	19.03	
CsFAPI/AKC	K_0.05_Cs_0.1_FA_0.85_PbI_3_	KCl + NH_4_Cl	Reverse	1.022	25.03	78.73	20.15	3
			Forward	1.016	24.96	76.92	19.51	
RbCsFAPI	Rb_0.05_Cs_0.1_FA_0.85_PbI_3_	No	Reverse	1.017	24.49	76.12	18.96	24
			Forward	1.006	24.36	58.89	14.43	
RbCsFAPI /KIAC	K_0.05_Rb_0.05_Cs_0.1_ FA_0.8_PbI_3_	KI + NH_4_Cl	Reverse	1.083	24.79	78.61	21.10	4
			Forward	1.072	24.66	76.74	20.29	
MAPI	MAPbI_3_	No	Reverse	1.037	21.83	76.32	17.25	17
			Forward	1.001	21.81	65.24	14.24	
MAPI /Au_NPs	MAPbI_3_	Au_NPs^b^	Reverse	1.059	23.37	76.81	19.01	16
			Forward	1.047	23.11	66.12	16.02	
FAPI	FAPbI_3_	No	Reverse	0.915	23.45	66.76	14.32	29
			Forward	0.859	23.18	51.23	10.21	
FAPI /MACl	FA_1-x_ MA_x_PbI_3_	MACl	Reverse	1.008	24.71	80.36	20.01	2
			Forward	1.003	24.69	78.58	19.46	

Perovskite composition, additive, *J-V* curve parameters, PCE and hysteresis index of champion solar cells. No perovskite surface treatment.

^a^Hysteresis Index, noted HI, defined as (PCE_Rev_ − PCE_For_)*100/PCE_Rev_.

^b^Au_NPs: Gold Nanoparticles.

Supplementary Fig. 3 are SEM top views of the films. The CsFAPI sample is characterized by the presence of pinholes, while the other films well-cover the substrate. We observe that MACl and NH_4_Cl additives favor the formation of large grains. It is also the case when gold nanoparticles are added in the PPS. Further information on the final film morphologies have been extracted from the cross-sectional views (Fig. 1c–k). Only the CsFAPI layer presented voids embedded at the perovskite/mesoporous TiO_2_ interface. The series of perovskite films on the right, namely CsFAPI/AKC, RbCsFAPI/KIAC, MAPI/Au_NPs and FAPI/MACl were prepared in the presence of additives. The very interesting point is that all these layers presented large grains, a monolithic structure, vertical grain boundaries and perfect contacts with the charge transporting layers (Supplementary Fig. 3j–m).

In the following, both (i) the effect of PPS spinning and anti-solvent dripping, and (ii) the effect of thermal annealing have been thoroughly investigated by the GD-OES technique completed by XRD measurements. The sputtering plasma, and then the probed sample surface, was delimited by a O-ring of 4 mm in diameter, which is close to the final solar cell size. Figure 1b shows the final crater generated in a film (top) and in a full PV device (bottom) by the plasma beam after their entire thickness analysis by GD-OES. The precursor and MHP layers deposited on TiO_2_ were quickly analyzed for the various preparation steps since the delay between the end of the preparation and the starting of the GD-OES measurement was less than 60 s while the GD-OES measurement lasted 35 s. The elemental depth distribution of the MHP film’s constituents was recorded at each stage of the film preparation process.

### Spin coating and anti-solvent dripping processes

The spin coating routine comprised a first stage of spinning at low speed (typically at 1000 rpm) to spread uniformly the PPS on the surface and then a stage at higher speed (typically at 4000 rpm) to start the film formation and solvent elimination. The dripping of anti-solvent was done at an accurate time which was carefully optimized for each system. Our aim was to track and understand the distribution of perovskite precursor (represented by the profile of Pb) and solvent (represented by the profile of the S element contained in DMSO) upon the spin coating step. This section focuses on GD-OES measurements done upon the CsFAPI/AKC perovskite preparation since the final S and Pb profiles were close after the spin coating step for all the various investigated samples (Supplementary Fig. 4). The results of S and Pb profiles without (W/O) and with chlorobenzene (CB) dripping are presented in Fig. 2a–f.

The depth profiles of Pb and S are shown in Fig. 2a–c and Fig. 2d–f respectively. Two distinct layers are clearly visible, differing by the intensity of S and Pb signals. The first part of the profile represents the upper layer, and the second part represents the lower layer since deeper part of the film is probed with increasing GD-OES sputtering time. The interface between the two layers, defined as the slope sign change of the curves (Supplementary Fig. 5) is shown as a vertical dashed line. One could note that the interface position does not move significantly. Considering similar erosion rate inside the layer, the similar erosion time indicates similar thickness of the upper layer for all MHP. When the spin coating time reached 25 s without dripping, two Pb peaks with the same intensity were visible in the profile (Fig. 2a). The film is then made of a colloidal dispersion of precursor aggregates and solvent^44–46^. Considering constant emission yield, similar intensity indicates similar quantity of eroded Pb. After 40 s of spin coating, so at the end of the whole spin coating routine, without dripping, the decrease of the first peak was accompanied by the increase of the second one. It means that parts of the upper layer aggregates which contain Pb was transferred to the lower layer upon spinning but the interface between the layers did not move significantly (Fig. 2a). The movements of S and Pb were opposite. In Fig. 2d, with the increase of the spin coating time, the solvent amount, traced by S, in the lower layer was significantly reduced but remained near constant in the upper layer. The explanation is twofold: (1) The free solvent of the lower layer is transferred to the upper layer during continuous spinning, and the solvent from the upper layer is thrown out due to the centrifugal force and evaporation under the high-speed operation of the spin coater; (2) Continuous downward movement of Pb during spin coating process causes S to move upward. Another finding is that with the increase of spin coating time, the first peak of the S profile, which is representative of the upper layer, has moved to the right, which indicates that the solvent on the most surface is quickly thrown out under the action of centrifugal force and evaporation.

In the case WITH CB dripping at 25 s. (Fig. 2c), only one broad Pb peak is visible at 25 s. At the end (40 s), the height of the peak increases and the peak maximum is shifted downward. The use of CB induces, a loss of solvent, the formation of a gel and the nucleation of what is described in the literature as a solvent-complex crystalline phase. This phase results from the interaction of DMSO with the precursor solutes. The intermediate phase is formed due to the O-donor Lewis base properties of DMSO. Its strong Lewis basicity favors the formation of a phase(s) with AX, PbI_2_, KCl, NH_4_Cl…^47,48^. For instance, in the case of the well-investigated MAPbI_3_ perovskite, this crystalline phase is escribed as MA_2_Pb_3_I_8_•2DMSO^49,50^. We can also mention that NH_4_^+^ is also a Lewis base that can presumably also interact with the precursors. The crystal phase formation enhances the measured performance of Pb. To better illustrate the role of CB in the preparation process, Fig. 2b juxtaposes the depth profiles of Pb for the samples spin coated for 25 s with and W/O CB. The presence of a single broad peak with strong intensity in place of two less intensive peaks can be interpreted as an acceleration of the solvent-complex phase formation by CB. Moreover, this peak (green curve in Fig. 2b) is very similar to that W/O CB spin coating for 40 s (blue curve in Fig. 2a), except that the peak with CB spin coating for 25 s is broader. It is concluded that the MHP obtained from W/O CB spin coating for 40 s is like that obtained with CB spin coating for 25 s. In Fig. 2f, the S profile from the sample WITH CB shows the same trend as in Fig. 2d. However, the difference is that the intensities of the S curve W/O CB are higher than that WITH CB at 25 s (Fig. 2e). It means that the addition of CB during spin coating accelerates the solvent removal process, causes a rapid supersaturation and then promotes the precipitation of the crystalline solvent-complex phase. This is clearly seen on the XRD patterns without and with CB in Supplementary Fig. 7. If XRD shows a crystallized precursor phase without CB dripping (peak at 11.79°), the intensity of the peak is much higher with CB while the diffraction peak is shifted to 11.91°, maybe because of a slightly different composition.

To better understand the above processes, we have developed a quantitative approach based on the curve integration that has allowed us to distinguish the upper and lower layers. Our approach is summarized and exemplified in Supplementary Fig. 5. In this approach, an area under the elemental depth profile curve in the zone, selected as the layer, is associated with the total (integral) quantity of the element in the layer. So, the evolution of the total content of the solvent and the precursor inside each layer and the contribution of each layer as a function of the treatment can be surveyed. In Fig. 2g, we can see that the total area of peak integral of Pb increases with the treatment time from 25 to 40 s spin coating. Whether it is spin coating for 25 s or 40 s, the total integrated Pb area with CB is always greater than the total integrated Pb area W/O CB. It proves that CB can promote the formation of perovskite precursor crystals. The relative proportions of the upper layer and the lower layer inside the whole profile, indicated by percentage in Fig. 2g, show that the proportion of Pb in the lower layer increased as the spin coating time increased. While CB favors the solvent-complex phase formation, it also accelerates the removal of the free solvent. In Fig. 2h, we observe that the total quantity of S was greatly reduced with treatment time, which proves that no matter adding CB or increasing the spin coating time, the solvent content is reduced. However, as analyzed above, the overall proportions shown by percentages in Fig. 2h confirm that the free solvent is gradually transferred to the upper layer with increasing spin coating time.

**Fig. 3:**
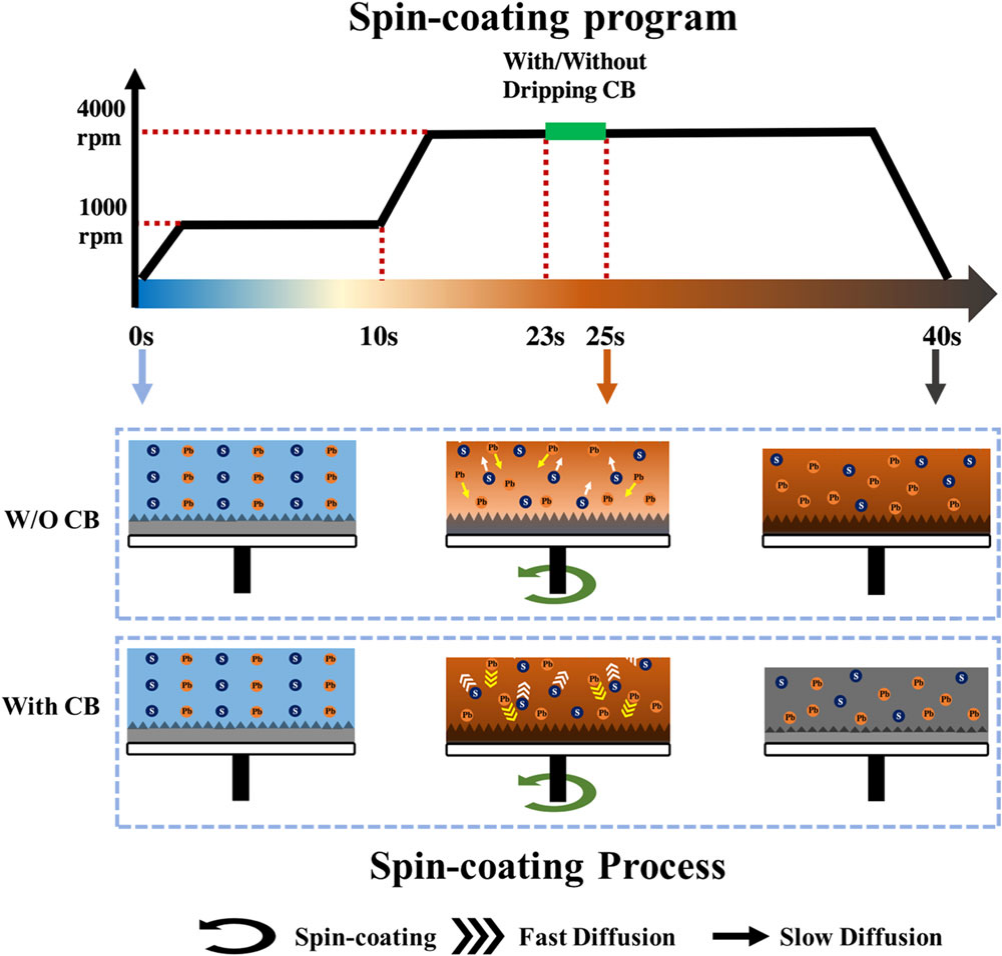
Element redistribution upon spin coating. (Top) Spin-coating routine. (Bottom) Schematic of distribution of Pb and S elements observed for 0 s, 25 s, and 40 s spin-coating times, without and with CB dripping.

According to our above systematic analysis, the whole process of spin coating is summarized in Fig. 3. The joint action of dripping CB and prolonged spin coating time results in an excess of solvent in the upper layer when the spin coating process is completed. On the contrary, the precursor crystallization is higher in the lower layer than in the upper layer and it results in an enrichment in lead. The addition of CB accelerates the transfer speed of Pb in the film and promotes the precursor phase formation. This is a general rule for the spin coating step of perovskite layers preparation.

### Annealing and (re)crystallization process

**Fig. 4:**
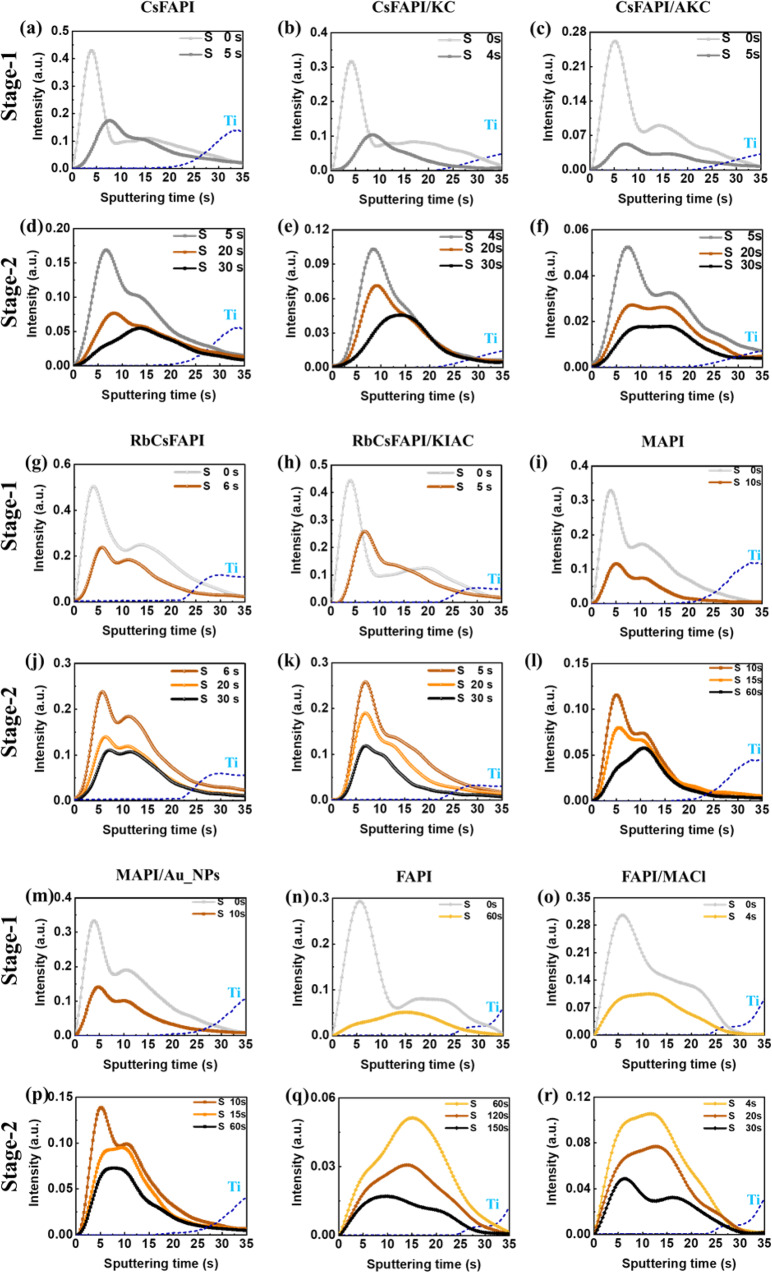
Evolution of GD-OES sulfur element (S) profile upon thermal annealing. Various perovskite precursor layers are followed upon Stage-1 and Stage-2 of thermal annealing. **a** CsFAPI at stage-1. **b** CsFAP/KC at stage-1. **c** CsFAPI/AKC at stage-1. **d** CsFAPI at stage-2. **e** CsFAP/KC at stage-2. **f** CsFAPI/AKC at stage-2. **g** RbCsFAPI at stage-1. **h** RbCsFAPI/KIAC at stage-1. **i** MAPI at stage-1. **j** RbCsFAPI at stage-2. **k** RbCsFAPI/KIAC at stage-2. **l** MAPI at stage-2. **m** MAPI/Au_NPs at stage-1. **n** FAPI at stage-1. **o** FAPI/MACl at stage-1. **p** MAPI/Au_NPs at stage-2. **q** FAPI at stage-2. **r** FAPI/MACl at stage-2. (Dashed line in **a**–**r**): Ti profile corresponding to the *meso*-TiO_2_). Source data are provided as a Source Data file.

The elimination of residual solvent, which is partly complexed to the perovskite precursors, is a key occurrence upon the MHP film crystallization and growth. We have used the GD-OES technique to follow the depth profile evolution of the solvent leaving perovskite films by evaporation upon thermal annealing. Indeed, the solvent employed was DMSO or DMF-DMSO mixtures, with DMF being eliminated in a great extent by the dripping performed upon the spin coating step^45,51,52^. By GD-OES, we have tracked the S element contained in DMSO. As a strongly coordinating solvent^53^, the DMSO content was quite high in the initial layer. The S element profile for the various films and various annealing times are presented in Figs. 4a–r. With increasing sputtering time, deeper part of the precursor/MHP layer was analyzed until reaching the *meso*-TiO_2_ layer (dashed black line in Figs. 4a–r). Before annealing, the DMSO profile was asymmetrical in all kinds of samples, and we observed that the outer part of the film was richer in solvent than the inner part. For the annealing, our results distinguished two stages. **Stage-1** corresponded to the initial film color change from yellow translucent to brown (Supplementary Fig. 6). XRD patterns show the formation and growth beginning of the α-phase (perovskite dark phase) during this stage (Supplementary Figs. 7–10). This phase was initially absent in all the samples except in the FAPI/MACl one. The most superficial residual solvent evaporated then for all kinds of perovskites (Fig. 4a–c, Fig. 4g, i, and Fig. 4m–o). This stage varied from <4 s to 60 s, depending on the PPS composition and then on the final perovskite composition as summarized in Supplementary Table 2. We observed that it was longer (10 s) for the DMSO-rich MAPI precursor film (also annealed for 1 h) and very long (60 s) for the FAPI film which corresponds to a poorly stable compound in its perovskite α-phase^54^. **Stage-2** corresponded to the brown to dark brown color change. The XRD patterns (Supplementary Figs. 7, 8 and 10) reveal the presence of an initial crystal phase formed upon the spin coating for the CsFAPI, RbCsFAPI, and FAPI systems. It is attributed to solvent-complex crystalline phases except for FAPI/MACl where MACl favors the nucleation of the α-phase. These phases were decomposed to the perovskite one upon thermal annealing. The structural solvent was evaporated from the complexes and crystal growth occurred then. The MAPI precursor layers with and without Au_NPs (Supplementary Fig. 9) were initially poorly crystallized but the same process occurred.

In all cases, the solvent was mostly eliminated after 2 or 4 min (Supplementary Fig. 11, 12) even if the full optimized annealing times were longer (Supplementary Table 2).

**Fig. 5:**
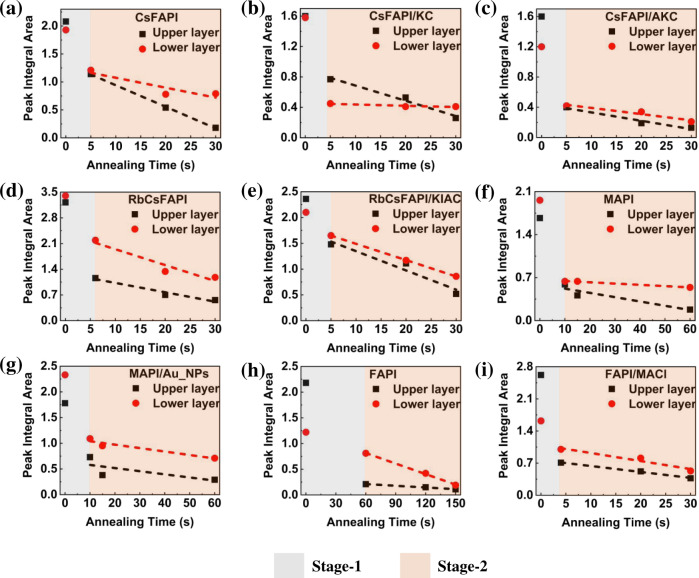
Solvent evolution tracking in the upper and lower layers. Peak integral area of the GD-OES profiles of Fig. 4 of upper layer and lower layer at different annealing times. **a** CsFAPI. **b** CsFAP/KC. **c** CsFAPI/AKC. **d** RbCsFAPI. **e** RbCsFAPI/KIAC. **f** MAPI. **g** MAPI/Au_NPs. **h** FAPI. **i** FAPI/MACl. (Black dashed line: linear fit line of upper layer in stage-2; Red dashed line: linear fit line of lower layer in stage-2). Source data are provided as a Source Data file.

From the results reported in Fig. 4, we observed an apparent strong effect of the additive on the solvent distribution upon annealing. However, to refine the analysis, we developed quantitative tools for a sound S change investigation. We divided the layer into an upper part and a lower part as described above (see also Supplementary Fig. 5) and we report in Fig. 5 the integral of each layer at various annealing times. They confirm the fast and large solvent elimination upon **Stage-1** for both the upper and the lower layers. Moreover, XRD patterns (Supplementary Figs. 7–10) show that, for all the samples, the initial phase is transformed into the perovskite one during this initial period and it explains the films color change. Only the FAPI/MACl sample contained predominantly the α-phase after spin coating (as-cast sample) (Supplementary Fig. 10) and the **Stage-1** duration was then very short.

Based on the above analysis and XRD data, we determined that the recrystallization occurred in Stage-1 (grey zone in Fig. 5) and that film growth mainly occurred in Stage-2 (red zone in Fig. 5). In this stage, we observed solvent elimination at about a constant speed, and we determined the slope of the two linear fits: SUS-2 slope for the upper layer and SLS-2 slope for the lower layer. Then, we defined two parameter indexes related to the relative growth speed of each layer. The first one is GI for Gap Index defined as (SUS-2 − SLS-2)*100/Max{SUS-2,SLS-2}. SUS-2 and SLS-2 are reported in Table 2. Through the analysis and comparison of the slopes of the two curves (Table 2), the films’ growth direction could be clearly judged for the different perovskites. We introduce GI as the main parameter to define the difference in film growth among perovskites (Table 2).

**Table 2:** Slope parameters for various precursor solution compositions

System	Additive	SUS-2^a^	SLS-2^b^	GI^c^ [%]	Growth direction^d^
CsFAPI	/	−0.0392	−0.0183	+53.3	D
	KCl	−0.0192	−0.0024	+87.5	D
	KCl + NH_4_Cl	−0.0113	−0.0082	+27.4	L
RbCsFAPI	/	−0.0266	−0.0434	−38.7	U
	KI + NH_4_Cl	−0.0371	−0.0323	+12.9	L
MAPI	/	−0.0072	−0.0024	+66.7	D
	Au_NPs	−0.0061	−0.0072	−15.2	L
FAPI	/	−0.0013	−0.0078	−83.3	U
	MACl	−0.0135	−0.0176	−23.3	L

They are extracted from the linear fit lines of upper layer and lower layers at stage-2.

^a^SUS-2: The Slope of Upper layer in the Stage-2.

^b^SLS-2: The Slope of Lower layer in the Stage-2.

^c^GI (Gap Index) = (SUS-2 − SLS-2)*100/Max{SUS-2,SLS-2}.

^d^*D* downward, *L* lateral, *U* upward.

Figure 5 and Table 2 provide us the following three conclusions: (i) When SUS-2 is greater than SLS-2, the film grows downward (from top to bottom). These samples exhibit a large GI, higher than +30%. A downward growth is found for CsFAPI, CsFAPI/KC, and MAPI films. (ii) If SLS-2 is greater than SUS-2 with GI lower than −30%, the film grows upward (from bottom to top). This is the case of the RbCsFAPI and FAPI samples. (iii) Finally, when the slope of the upper layer in the stage-2 (SUS-2) is very close to that of the lower layer in the stage-2 (SLS-2), it results in more homogeneous films with big monolithic grains. We define this growth trend as lateral growth. The GI of the samples that meet the lateral growth falls between +30% and −30% (Table 2). This case is encountered for the CsFAPI/AKC, RbCsFAPI/KIAC, MAPI/Au_NPs, and FAPI/MACl samples, those which exhibit a monolithic structure in Fig. 1. (i) and (ii) cases lead to multiple and oblique grain boundaries and to the production of rather small perovskite grains (Fig. 1 and Supplementary Fig. 3). Films of (i) and (ii) cases have also a higher roughness compared to layers stemming from a lateral growth ((iii) case) (Supplementary Fig. 13).

In the cross-sectional SEM view of the CsFAPI sample (Fig. 1c), we found the appearance of voids embedded at the perovskite/TiO_2_ interface and pinholes on top views. To deeply understand the causes of this phenomenon, we introduce another essential parameter, MLU, for the Multiple of Lower layer residual solvent ratio over Upper layer residual solvent ratio at the critical annealing times reported in Table 3. In Table 3 and Supplementary Fig. 14, we can see that the MLU for the CsFAPI, at 4.45, is the highest among all kinds of perovskites we investigated. This means that after 30 s of annealing time, the Lower layer RSR is 4.45 times higher than the Upper layer RSR. In other words, the CsFAPI sample is the only one for which the inner solvent is badly eliminated. We can then suppose that, due to the formation of a perovskite crust at the film surface, solvent molecules remain entrapped in the inner part of the layer^55^. Voids are formed upon solvent escaping, releasing a poor *meso*-TiO_2_/perovskite interface. In the cross-sectional views of other samples, no voids were present. It can be seen from the MLU value that the solvent will not be entrapped as long as this value is not too high, i.e., MLU ≤ 3.0 according to Table 3.

**Table 3:** Analysis of the residual solvent

System	Additive	Annealing time [s]	Upper layer RSR^a^ [%]	Lower layer RSR^b^ [%]	MLU^c^
CsFAPI	/	30	18.35	81.65	4.45
	KCl	30	37.62	62.38	1.65
	KCl + NH_4_Cl	30	38.16	61.84	1.62
RbCsFAPI	/	30	32.17	67.83	2.10
	KI + NH_4_Cl	30	37.66	62.34	1.65
MAPI	/	60	24.94	75.06	3.00
	Au_NPs	60	32.27	67.73	2.09
FAPI	/	150	38.63	61.37	1.58
	MACl	30	41.08	58.92	1.43

Relative proportion of upper layer RSR and lower layer RSR at late Stage-2.

^a^Upper layer RSR (Residual Solvent Ratio) = Integral area of upper layer/(Integral area of upper layer + Integral area of lower layer).

^b^Lower layer RSR = Integral area of lower layer/(Integral area of upper layer + Integral area of lower layer).

^c^MLU (Multiple of Lower layer RSR over Upper layer RSR) = Lower layer RSR/Upper layer RSR.

Since the properties of the initial layer stemming from the spin coating/anti-solvent stream step are essential for the recrystallization and growth of the perovskite layers, we have also investigated the effect of the chemical nature of the anti-solvent taking the example of FAPI/MACl layers. Supplementary Fig. 15 compares the GD-OES solvent profiles for precursor layers prepared with chlorobenzene and with diethyl ether (DEE) anti-solvents. In both cases, the initial layer presented a DMSO excess near the surface. Interestingly, with diethyl ether, the intensity of the S-signal was lower, and DEE acts as a better dryer of the layer. The outer DMSO was quickly eliminated during the first seconds of thermal annealing in both cases. Another interesting point is that a more homogeneous elimination of DMSO subsequently occurred upon annealing in the case of DEE. It resulted in a lower GI value (−10.2% instead of −23.3%). We can conclude that the type of anti-solvent affects the growth parameters of the layer and is an important lever to tune the final properties of the perovskite film. We also note that, in the present case, it did not change the layer growth direction.

### Perovskite layers growth models and types

**Fig. 6:**
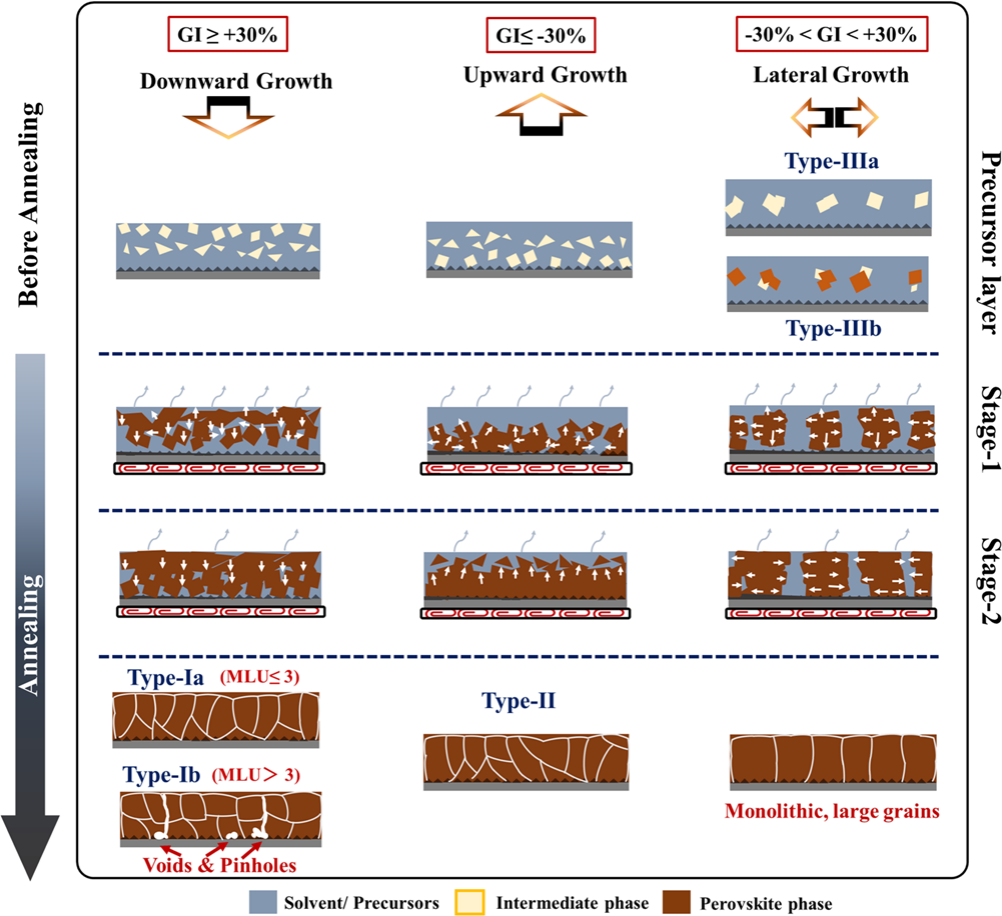
Parameters and typologies of perovskite layers growth models. Perovskite film formation process upon annealing. Typology of perovskite films growth: Grain growth direction, mechanism, and growth parameters. The white arrows point to the growth directions.

The large amount of information on the perovskite films growth stages and mechanism upon thermal annealing which have been gathered in the present work have been schematically summarized in Fig. 6. We have evidenced two stages. Upon **Stage-1**, the crystallites combining the solvent molecules-cations-lead-halogens are thermally decomposed and the solvent molecules evaporate. By a rearrangement and recrystallization process, perovskite crystals are formed which grow in all the directions. First, our findings show that the location and density of the first seeds/crystallites formed then, and which is a consequence of the microstructure and composition of the precursor layer, will decide the subsequent steps, especially the layer growth direction as illustrated in the middle part of Fig. 6. Their concentration at the top, middle and bottom part of the layer will drive the growth in the downward, lateral, and upward directions, respectively. Moreover, their density will decide the final size of the crystals in the perovskite layer. Sparsely dispersed crystallites are targeted since it will lead to the growth of large grains which are beneficial for the final performances of the devices. Upon **Stage-2**, different behaviors have been found depending on the PPS composition. Our study shows that the additives govern the film formation since the early stages and have a crucial role to tune the size and composition of the precursor aggregates and initial nuclei^44,56^. The gradual evaporation of MACl or NH_4_Cl can self-regulate the perovskite crystal growth and favor a slow conversion^57^. In general, additives will promote the lower nucleation site density, bigger precursor crystallites, control the solubility of the precursor solutes and the composition of the crystallized intermediate phases, drive the solvent elimination speed and then the layer growth speed. During **Stage-2**, a transition to a directional growth of the perovskite grain occurs (Fig. 6). The direction and speed of the growth will define the final MHP layers properties.

Finally, the present comprehensive study allows us to propose a typology of the perovskite film growth based on the crystal growth direction as shown in Fig. 6. **Type I** is encountered when the film grows downward. If the film is dense with a rather slow growth, the type is **Ia**. A special case is encountered when a surface crust is formed. The inner solvent is especially difficult to eliminate then and is entrapped in the inner part of the film. Its escape leads to the formation of voids at the bottom contact interface and of pinholes (**Type Ib**). The second case is when the growth direction is upward (**Type II**). Both **Type I** and **Type II** lead to rather small grains with multiple grain boundaries. Finally, the targeted growth behavior for high device efficiency, good homogeneity and good stability is the lateral one that we name **Type III**. We have noted that this type was encountered when the initial as-cast layer contained the perovskite phase (FAPI/MACl sample) (**Type IIIb**) or not (**Type IIIa**). For **Type III**, a monolithic structure with reduced and vertically oriented grain boundaries is formed. This growth mode results in smoother layers. It favors the fast charge transfer to the contact at both MHP film side in the solar cells. The corresponding relative growth parameters are spelled out in Fig. 6 and Table 4.

**Table 4:** Growth type as a function of the GI and MLU parameters

Type	Ia	Ib	II	III
GI	≥30%	≥30%	≤−30%	−30% <<30%
MLU	≤3	>3	≤3	≤3

## Discussion

We have employed the GD-OES technique, completed by X-ray diffraction and scanning electron microscopy observations for the study of spin coating, anti-solvent dropping and thermal annealing preparation steps of a large variety of PV halide perovskite thin films. GD-OES has allowed us to profile and track solvent and elements fates. We have distinguished the inner and outer parts of the layers. As a general law, prolonged spin coating time results in an excess of solvent in the upper layer part and in a lead accumulation in the inner part when the spin coating process is completed. This effect is accelerated by using an anti-solvent steam which also favors the solvent elimination. The anti-solvent treatment must be triggered when the layer is dry enough to reach the supersaturation under the effect of the anti-solvent stream and to induce the generation of crystalline intermediate phases of optimum size and density. Additives must also be used for a better optimization of these parameters. These additives, which are chloride and Lewis bases in the present work, enter in the composition of these intermediate.

For the thermal annealing, we have distinguished two stages. Upon **Stage-1**, solvent, in the outer film part, is eliminated. The active perovskite phase is formed by decomposition of the precursor phase, forming the initial perovskite crystallites which grow in all the space directions. During **Stage-2**, the perovskite grains grow in a preferential direction, and the residual solvent is eliminated. The initial composition of the intermediate is then key to regulate the grain growth speed and direction which has been tracked by how solvent is eliminated throughout the film thickness. We have proposed growth parameters indexes, namely, GI and MLU to quantify and accurately describe the differential solvent elimination between the inner and upper layer parts. They have allowed us to propose an universal new typology which distinguishes three types depending on the growth direction of perovskite crystals in thin films and which has been validated by a large number of various halide perovskite films studied: downward (**Type I**), upward (**Type II**) and lateral (**Type III**). The growth direction is key for the final properties of the films. Among them, the best efficiency has been always found for solar cells prepared with monolithic films resulting from a lateral growth mode. **Type III** growth mode must be targeted for the preparation of efficient perovskite films for solar cells.

## Methods

### Preparation of substrates, compact TiO_2_, and mesoporous TiO_2_ layers

Fluorine-doped SnO_2_ (FTO) substrates (TEC 7 from Pilkington) were etched pattern by zinc powder and 10% HCl solution prior to be cleaned with soap and water. The substrates were then immersed for 20 min in a concentrated 2.2 M NaOH in ethanol/water (10:1 v/v %) and rinsed with deionized water in an ultrasonic bath for 15 min. The substrates were subsequently heated at 500 °C for 15 min. The compact TiO_2_ hole blocking layer, noted *c-*TiO_2_, was prepared by aerosol spray pyrolysis. The TiO_2_ nanoparticle solution employed for the preparation of the mesoporous layer, noted *mp*-TiO_2_, was prepared in advance and stirred for at least 12 h. The TiO_2_ NR30-D paste (from Greatcell) was diluted in ethanol with a 1:7 w/w ratio. 45 μL of the solution was dropped on the *c-*TiO_2_ layer and spin coated at 2000 rpm for 15 s. The layer was then dried on a hotplate at 70 °C for 5-10 min and finally heated at 500 °C under an air flux for 30 min, cooled down to 200 °C and removed from the hotplate.

### Preparation of the PVK Layers

#### CsFAPI layers preparation

253.6 mg PbI_2_ (1.1 M, TCI), 77.4 mg FAI (Greatcell) and 13.0 mg CsI (TCI) were mixed in 400 μL DMF and 100 μL DMSO. The PPS was stirred for 2 h before use. The Spin coating program was 1000 rpm for 10 s and 4000 rpm for 30 s. One hundred microliters of chlorobenzene was dripped 23 s after starting of the spinning routine. The layers were finally annealed on a hotplate at 155 °C for 15 min^58^. These layers are denoted CsFAPI throughout the paper.

#### CsFAPI/KC layers preparation

253.6 mg PbI_2_ (1.1 M, TCI), 77.4 mg FAI (Greatcell), 13.0 mg CsI (TCI) and 3.5 mg KCl (Alfa Aesar) were mixed in 400 μL DMF and 100 μL DMSO. The PPS was stirred for 2 h before use. The spin coating program was 1000 rpm for 10 s and 4000 rpm for 30 s. 100 µL of chlorobenzene was dripped 23 s after starting of the spinning routine. The layers were finally annealed on a hotplate at 155 °C for 15 min. These layers are denoted CsFAPI/KC throughout the paper.

#### CsFAPI/AKC layers preparation

A mixed cation precursor solution with a 1.1 M concentration was prepared by mixing 78 mg of Formamidinium Iodide (FAI, greatcell), 253.6 mg of Lead Iodide (PbI_2_, TCI), 13 mg Cesium Iodide (CsI, TCI), 8 mg of Ammonium Chloride (NH_4_Cl, Alfa Aesar), and 1.9 mg of Potassium Chloride (KCl, Alfa Aesar) in 400 μL DMF and 100 μL DMSO. The solutions were stirred for a minimum of 7–8 h at 50 °C in a N_2_ glovebox. Thirty microliters of this solution was placed on top of the substrates. A two-step spin coating program was employed: first spinning at 1000 rpm for 10 s and then at 4000 rpm for 20 s. One hundred microliters of chlorobenzene was dripped 23–25 s after starting of the spinning routine. The films were then annealed at 155 °C for 20 min. These films and corresponding solar cells are noted CsFAPI/AKC.

#### RbCsFAPI layers preparation

73.1 mg of FAI (Greatcell), 5.3 mg of RbI (Alfa Aesar), 12.9 mg of CsI (TCI) and 253.6 mg of PbI_2_ (1.1 M, TCI) were mixed in 100 μL DMSO and 400 μL DMF. The PPS was stirred for a minimum of 2 h at room temperature in a N_2_ filled glovebox before use. Thirty microliters of this solution was placed on top of the substrates. A two-step spin coating program was employed: first spinning at 1000 rpm for 10 s and then at 4000 rpm for 30 s. One hundred microliters of chlorobenzene was dripped 23 s after starting of the spinning routine. The films were then annealed at 155 °C for 15 min in glovebox with N_2_ atmosphere. These layers are denoted RbCsFA throughout the chapter.

#### RbCsFAPI/KIAC layers preparation

A mixed alkali metal cation PPS was prepared by mixing 68.8 mg FAI (Greatcell), 2.1 mg KI (Sigma–Aldrich), 5.3 mg RbI (Alfa Aesar), 13.0 mg CsI (TCI), 8 mg NH_4_Cl (Alfa Aesar) and 253.6 mg of PbI_2_ (1.1 M, TCI) in 400 μL DMF and 100 μL DMSO. The solutions were stirred for a minimum of 4 h at room temperature in a nitrogen filled glovebox. 30 μL of this solution was placed on top of the substrates. A two-step spin coating program was ran: first spinning at 1000 rpm for 10 s and then at 4000 rpm for 30 s. One hundred microliters of chlorobenzene was dripped 23 s after starting of the spinning routine. The films were then annealed at 155 °C for 15 min. The PAI post-deposition treatment consisted in dropping 60 μL of a 4 mg/mL n-propylammonium chloride (PAI) solution onto the perovskite film after cooling. A one-step spin coating program was employed: 2000 rpm/s acceleration, 3000 rpm for 20 s. These layers are denoted RbCsFAPI/KIAC (no post-treatment) throughout the chapter.

#### MAPI layers preparation

The MAPbI_3_ precursor solution concentration was 1.35 M. 622.3 mg PbI_2_, 214.6 mg MAI were mixed in 1000 µL DMSO. The solution was stirred and kept warm at 60 °C for 2 h before use. The Spin coating program was 1000 rpm for 10 s and 4000 rpm for 30 s. One hundred microliters of chlorobenzene was dripped 23 s after starting of the spinning routine. The layers were finally annealed on a hotplate at 105 °C for 60 min. The best performances were achieved after the device preparation. These layers are denoted MAPI throughout the chapter.

#### MAPI/Au_NPs layers preparation

The MAPbI_3_ precursor solution concentration was 1.35 M. 622.3 mg PbI_2_, 214.6 mg MAI, and 15 µL of gold nanoparticles DMSO solution were mixed in 985 µL DMSO. The solution was stirred and kept warm at 60 °C for 2 h before use. The Spin coating program was 1000 rpm for 10 s and 4000 rpm for 30 s. One hundred microliters of chlorobenzene was dripped 23 s after starting of the spinning routine. The layers were finally annealed on a hotplate at 105 °C for 60 min. The best performances were achieved after the device preparation. These layers are denoted MAPI/Au_NPs throughout the chapter. The PAI post-treatment consisted of dropping 60 μL of a 4 mg/mL n-propylamine hydroiodide (PAI) solution onto the perovskite film after cooling. A one-step spin coating program was employed (2000 rpm/s acceleration, 3000 rpm for 20 s). After that, the sample did not need to be reheated.

#### FAPI layers preparation

276.6 mg PbI_2_ (1.2 M, TCI) and 103.2 mg FAI (Greatcell) were mixed in 400 μL DMF and 100 μL DMSO. The PPS was stirred for 2 h before use. The Spin coating program was 1000 rpm for 10 s and 4000 rpm for 30 s. 100 µL of chlorobenzene was dripped 23 s after starting of the spinning routine. The layers were finally annealed on a hotplate at 155 °C for 15 min. These layers are denoted FAPI throughout the chapter.

#### FAPI/MACl layers preparation

A mixed cation precursor solution with a 1.2 M concentration was prepared by mixing 103 mg of formamidinium iodide (FAI, Greatcell), 277 mg of PbI_2_ (TCI), and 19.5 mg of methylammonium chloride (MACl, Alfa Aesar) in 400 μL DMF and 100 μL DMSO. The solutions were stirred for a minimum of 2 h at room temperature in a nitrogen filled glovebox. Forty-five microliters of this solution was placed on top of the substrates. A two-step spin coating program was ran: first spinning at 1000 rpm for 10 s and then at 6000 rpm for 30 s. One hundred microliters of chlorobenzene was dripped 23 s after starting of the spinning routine. The films were then annealed at 155 °C for 13 min. The PEAI post-deposition treatment consisted in dropping 60 μL of a 10 mM 2-Phenylethylamine Hydroiodide (PEAI) solution (2.49 mg in 1 mL of isopropanol) onto the perovskite film after cooling. A one-step spin coating program was employed: 2000 rpm/s acceleration, 3000 rpm for 20 s. The best performances and lowest *HI* were achieved after 3–4 days of storage in the N_2_ filled glovebox. These not-treated FA_1-x_MA_x_PbI_3_ layers are denoted FAPI/MACl throughout the paper.

The hole transporting material (HTM) solution was prepared by dissolving 78 mg of Spiro-OMeTAD (Borun New Material Technology) in 1 mL of chlorobenzene. Then, 17.9 μL of bis(trifluoromethylsulfonyl)imide lithium salt solution (Li-TFSI) (Sigma–Aldrich) solution (517 mg in 1 mL ACN), 30.4 μL of TBP (tert-butylpyridine) (Sigma–Aldrich) and 14 μL of tris(2-1H-pyrazol-1-yl)−4-tert-butylpyridine)-cobalt(III) tris (bis(trifluoromethylsulfonyl)imide) (Dyesol, FK209) (376 mg in 1 mL acetonitrile) were added to this solution. Forty microliters of the HTM solution was spin coated at 4000 rpm for 30 s. Finally, the device was completed by thermally evaporating a 70–80 nm thick gold back contact on the Spiro-OMeTAD layer.

### Characterization methods

The structure of the organo-metal lead perovskite films was characterized by a PANanalytical X-Pert high-resolution X-ray diffractometer (XRD) operated at 40 kV and 45 mA and using the CuKα radiation with *λ* = 1.5406 Å. The morphology of perovskite thin films was measured using a field-emission SEM equipment (Zeiss Supra 40) in the in-lens mode.

Glow-Discharge Optical Emission Spectrometry (GD-OES) analyses were performed using a HORIBA Jobin Yvon GD Profiler 2 with Quantum Software. The instrument configuration included a RF-generator (at 13.56 MHz), a standard HORIBA Jobin Yvon glow discharge source with a cylindrical anode of 4 mm internal diameter and two optical spectrometers (a polychromator and a monochromator) for fast-optical detection. No loss in time resolution was ensured thanks to the detection system, composed of independent photomultiplicators (PM), which guarantee simultaneous detection of the signals corresponding to multiple wavelength with spectral resolution of 12 pm. The emission signals of 47 elements were recorded but only the signals of the species of interest are discussed in the paper. The selected wavelength for Pb and S were 220.357 and 180.738 nm, respectively. The optical system was purged by high purity N_2_ in order to detect emission lines in the VUV region. Prior to each analysis, the source was cleaned by sputtering a sacrificial Si wafer for 60 s. The interest of the technique is its rapidity and easiness for use. It does not need high vacuum nor specific sample preparation. Therefore, the films could be characterized rapidly (less than 1 min) after each step of preparation. High purity argon (99.999% minimum purity) was employed as the discharge gas. The Ar plasma was generated at an Ar pressure of 420 Pa and an applied power of 17 W. The precursor or perovskite layer was mounted on an O-ring at one side of the plasma chamber and used as a cathode. The total emission intensity depends on the erosion rate and emission yield. The data treatment and analysis were made in the hypothesis of a constant emission yield. Taking into account that the erosion rate can vary with chemical composition and microstructure inside the layer and between the layers, all the signals were normalized by the total emitted light. Only the normalized signals are shown and discussed in the paper.

The *J-V* curves were recorded by a Keithley 2410 digital sourcemeter, using a 0.1 V.s^−1^ voltage scan rate. The solar cells were illuminated with a solar simulator (Abet Technology Sun 2000) filtered to mimic AM 1.5 G conditions (100 mW/cm^2^). The illuminated surface was delimited by a black mask with an aperture diameter of 3 mm. The power density was calibrated at 100 mW.cm^−2^ by the use of a reference silicon solar cell^11^.

## Supplementary information

Supplementary Information

## Data Availability

Additional information related to this work is available from the corresponding authors on request. Source data are provided with this paper.

## Source data

Source Data
