# Supplementary material for: Control of perovskite film crystallization and growth direction to target homogeneous monolithic structures
Source: Nat Commun. 2022 Nov 4;13:6655. doi: 10.1038/s41467-022-34332-3 (PMC9636165; doi:10.1038/s41467-022-34332-3)
Supplement: Supplementary file 1 — Supplementary Information [file 41467_2022_34332_MOESM1_ESM.pdf]

Supplementary Information

Control of Perovskite Film Crystallization and Growth Direction to Target Homogeneous Monolithic Structures

Zheng et al.

Supplementary Table 1: Champion solar cells characteristics. *J-V* curve parameters, PCE and hysteresis index before and after post-treatment (capping layer).

Name	Capping layer	Scan direction	V_{oc} [V]	J_{sc} [mA. cm^{-2}]	*FF*	PCE [%]	*HI*[a)] [%]
CsFAPI/AKC	NO	Reverse	1.022	25.03	78.73	20.15	3
		Forward	1.016	24.96	76.92	19.51	
CsFAPI/AKC	YES (PAI)	Reverse	1.051	25.31	79.23	21.08	3
		Forward	1.049	25.02	77.91	20.45	
RbCsFA /KIAC	NO	Reverse	1.083	24.79	78.61	21.10	4
		Forward	1.072	24.66	76.74	20.29	
RbCsFA /KIAC	YES (PAI)	Reverse	1.121	24.86	80.83	22.53	4
		Forward	1.116	24.71	78.84	21.74	
MAPI /Au_NPs	NO	Reverse	1.059	23.37	76.81	19.01	16
		Forward	1.047	23.11	66.12	16.02	
MAPI /Au_NPs	YES (PAI)	Reverse	1.103	23.26	79.68	20.13	13
		Forward	1.091	23.14	70.42	17.78	
FAPI /MACl	NO	Reverse	1.008	24.71	80.36	20.01	2
		Forward	1.003	24.69	78.58	19.46	
FAPI /MACl	YES (PEAI)	Reverse	1.060	25.94	80.62	22.18	4
		Forward	1.052	25.93	78.26	21.35	

[a)] Hysteresis Index, noted HI, defined as $(PCE_{Rev}-PCE_{For})*100/PCE_{Rev}$

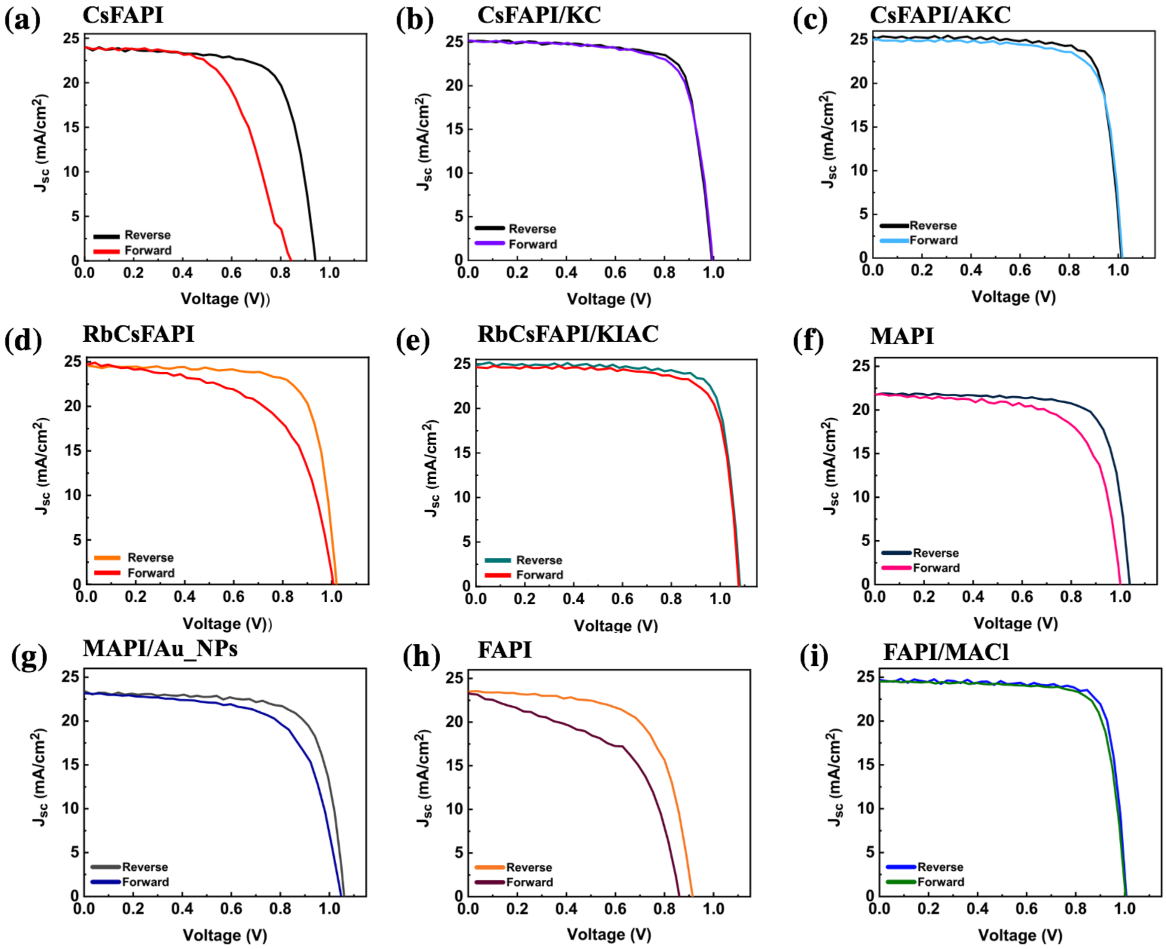

Supplementary Fig. 1: Typical *J-V* curves. Forward and reverse *J-V* curves of solar cells among the best investigated in the present work. (a) CsFAPI, (b) CsFAPI/KC, (c) CsFAPI/AKC, (d) RbCsFAPI, € RbCsFAPI/KIAC, (f) MAPI, (g) MAPI/Au_NPs, (h) FAPI and (i) FAPI/MACl. Source data are provided as a Source Data file.

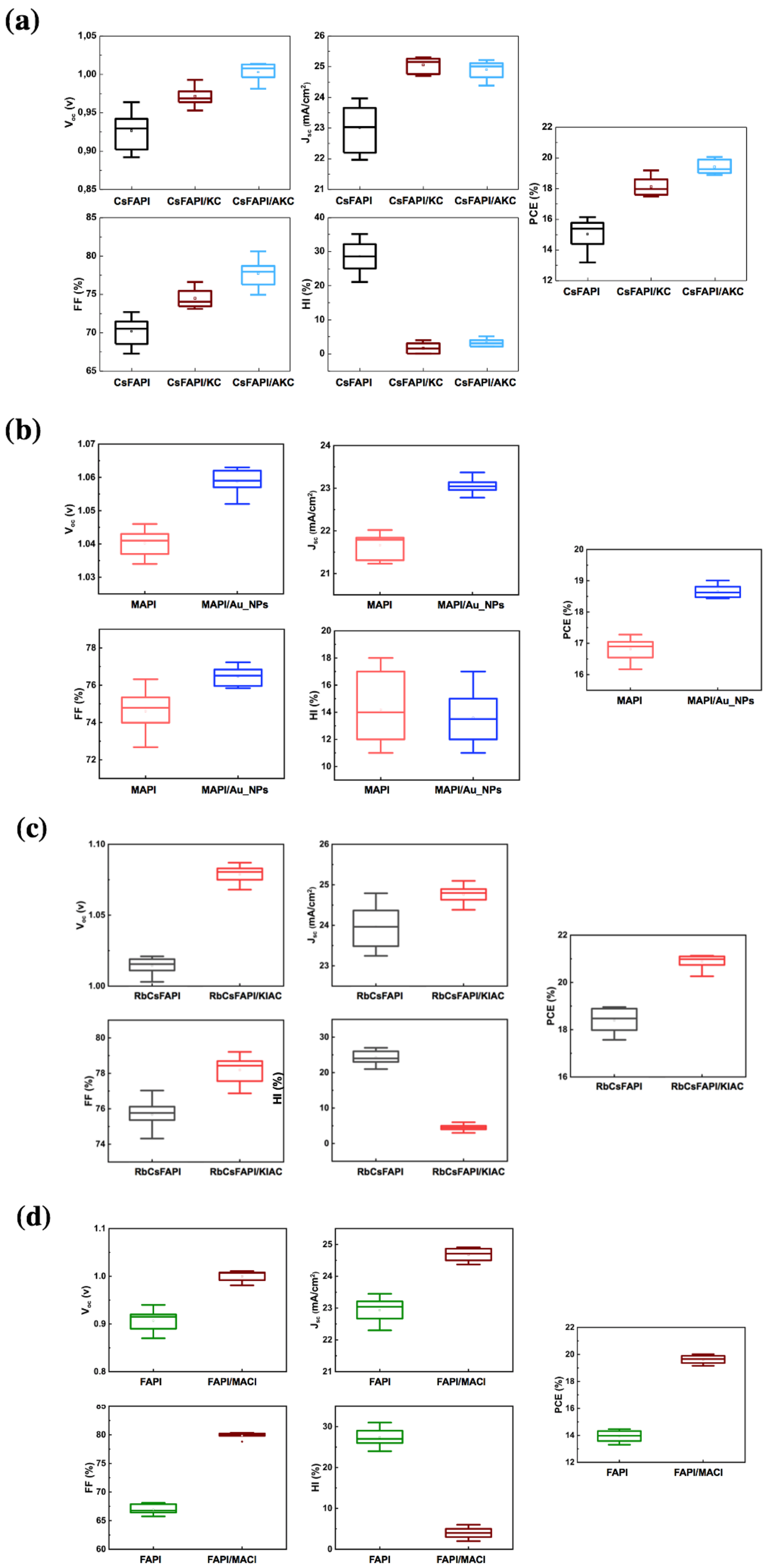

Supplementary Fig. 2: Statistics of *J-V* curve parameters. Based on 12 cells for each system. No perovskite surface treatment. (a) CsFAI system, (b) MAPI system, (c) RbScFAPI system and (d) FAPI system. Source data

are provided as a Source Data file

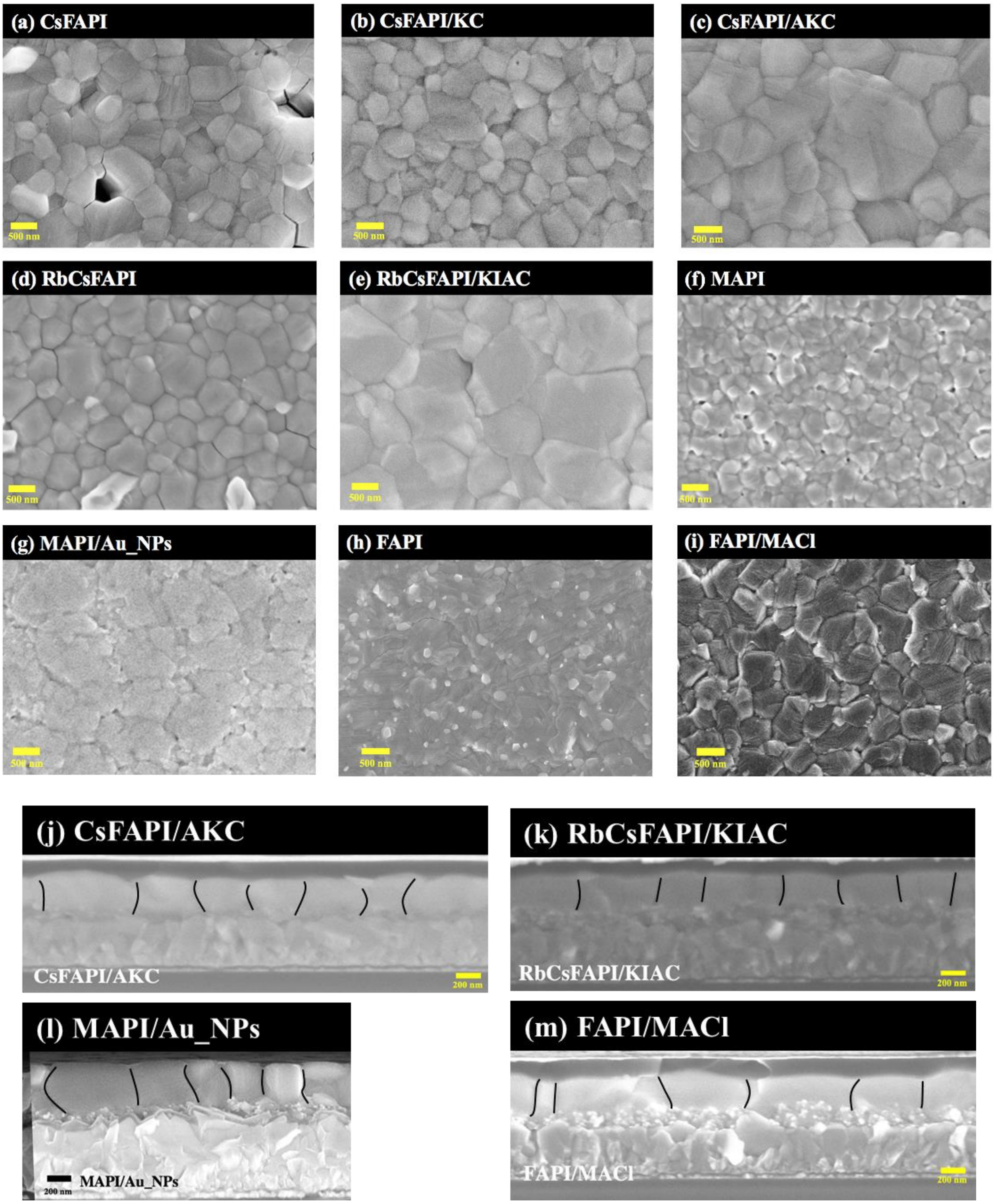

Supplementary Fig. 3: Perovskite SEM top and cross-sectional views. (a-c) CsFAPI based films: (a) pristine, (b) with KCl additive and (c) with KCl and NH_4Cl additives. (d,e) RbCsFAPI based films: (d) pristine and (e) with KI and NH_4Cl additives. (f) MAPI film and (g) MAPI with gold nanoparticles additives. (h) FAPI film and (i) FAPI film with MACl additive. (scale bar: 500 nm). (j-m) SEM cross-sectional views of monolithic structure with grain boundaries (scale bar 200 nm).

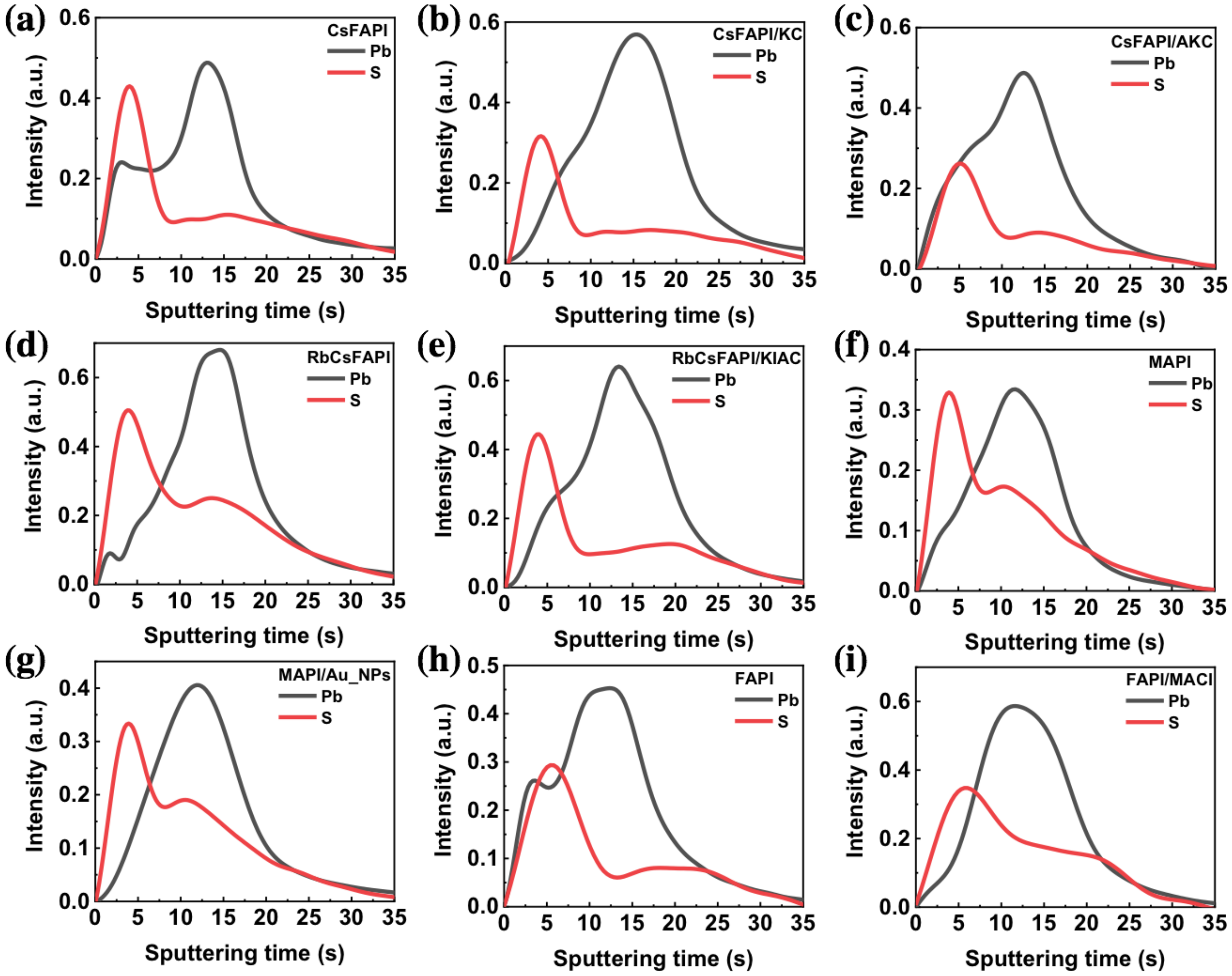

Supplementary Fig. 4: GD-OES profiles of S and Pb .GD-OES profiles after the PPS full spin-coating process with dripping for the various investigated samples. Source data are provided as a Source Data file.

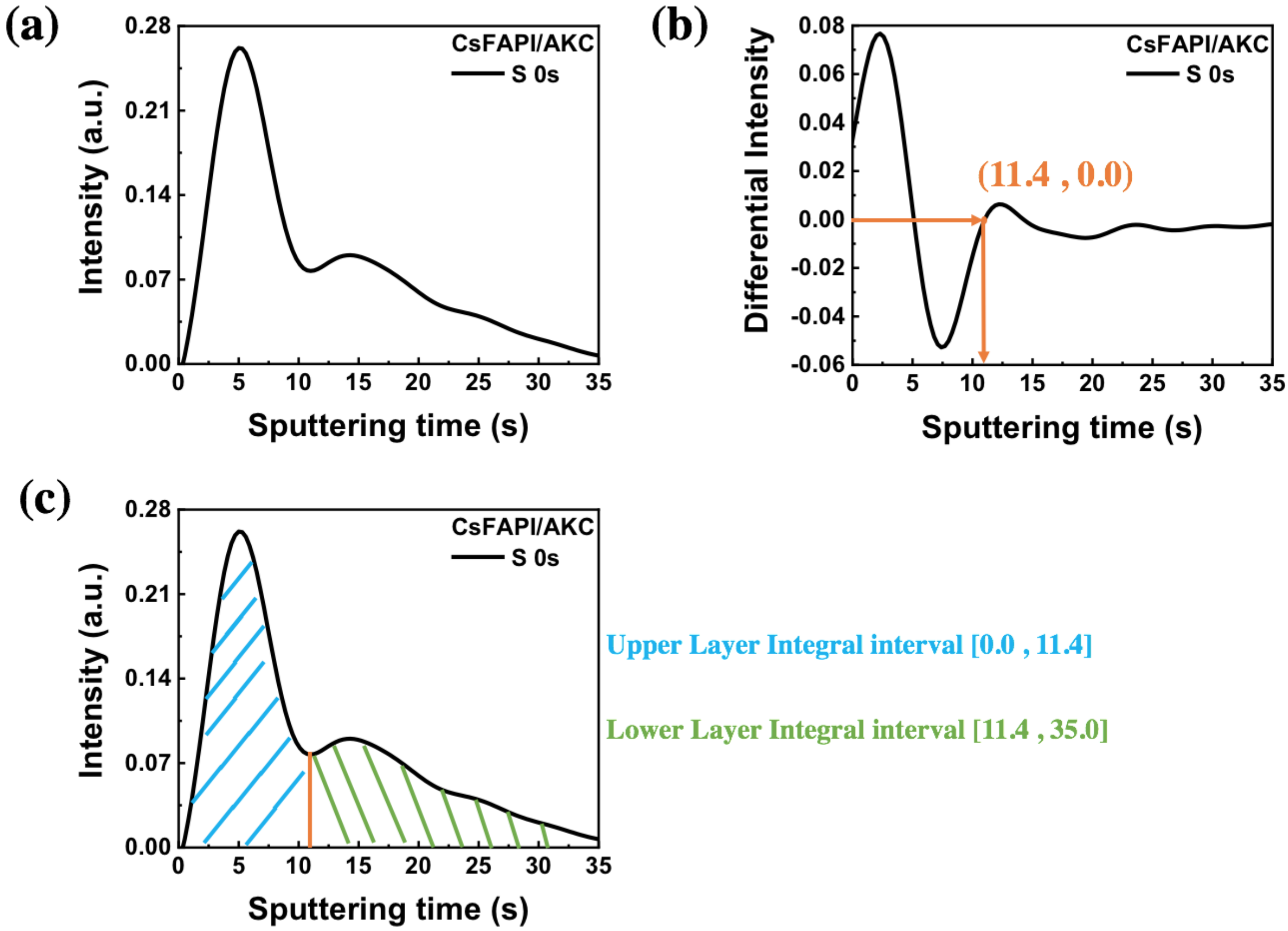

Supplementary Fig. 5: GD-OES profile analysis process. (a) Evolution of GD-OES sulphur element (S) profile in the CsFAPI/AKC perovskite precursor layer. (b) Differential data of (a). (c) Distribution of upper layer and lower layer in (a). Reprinted with permission from Supplementary Ref. [1]. Copyright 2022 John Wiley and Sons.

Comment: Supplementary Figure 5a shows the GD-OES profile of Sulphur (S) at 0 second. In order to find accurately the position of the first inflection point in **Supplementary Figure 5a**, we derived **Supplementary Figure 5a** to get **Supplementary Figure 5b**. In **Supplementary Figure 5b**, we found the exact inflection point coordinate at (11.4 , 0.0). It provided the time of the first inflection point at 11.4s. After determining this time, we divided **Supplementary Figure 5a** into two intervals for integration. The integral interval for the upper layer ranges from 0.0 s to 11.4 s (**Supplementary Figure 5c**). The integral interval for the lower layer ranges from 11.4 s to 35.0 s (**Supplementary Figure 5c**).

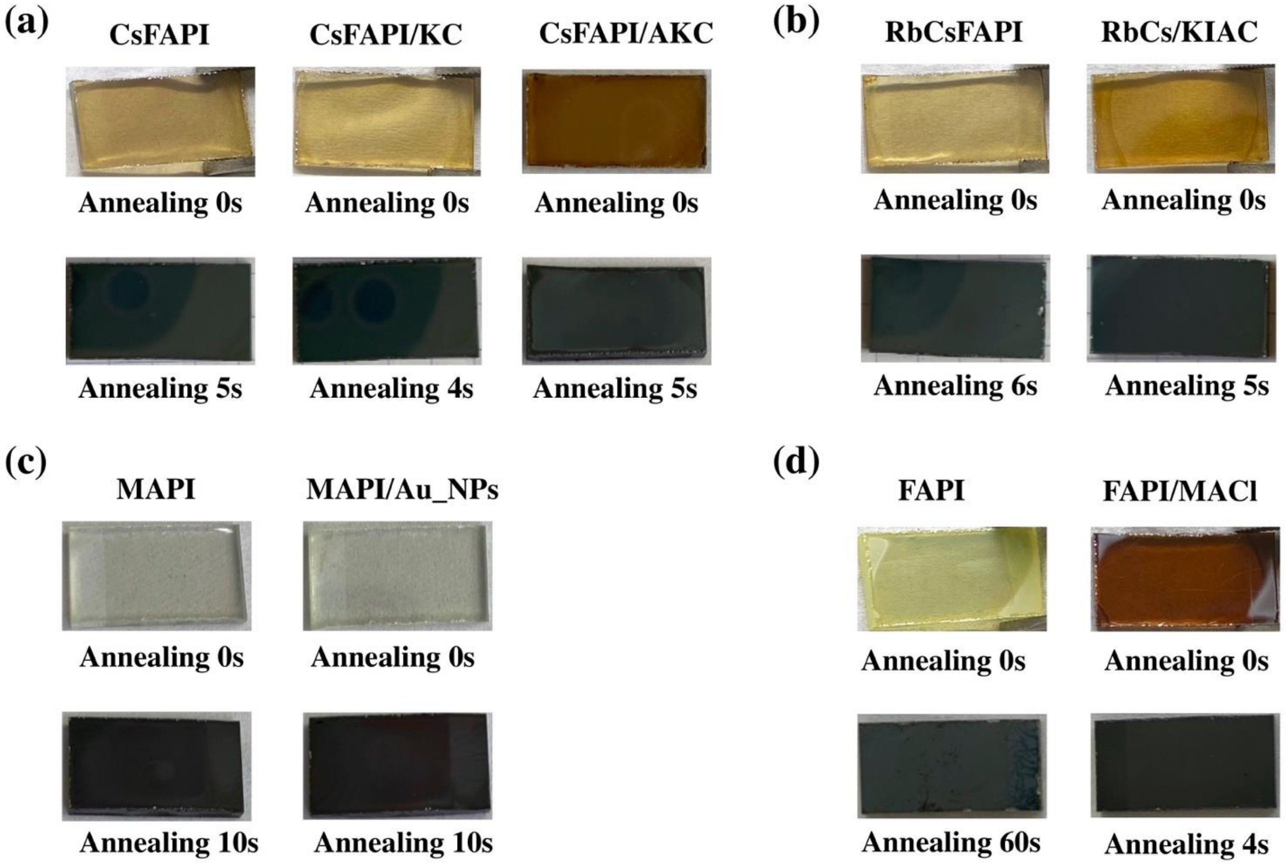

Supplementary Fig. 6: Color change upon Stage-1. (a) CsFAPI based film, (b) RbCsFAPI based film, (c) MAPI based film and (d) FAPI based film upon annealing on hotplate for several seconds.

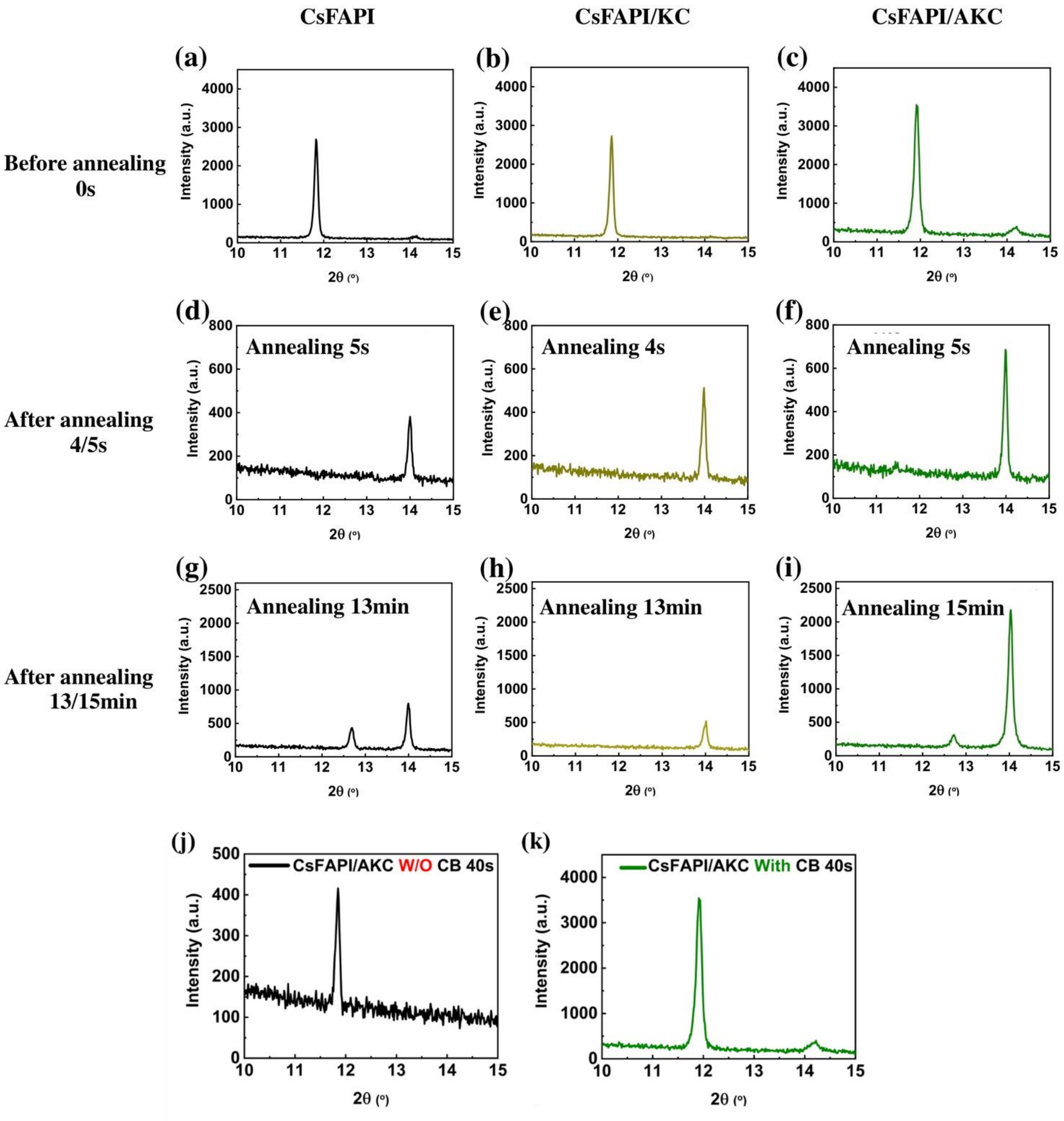

Supplementary Fig. 7: Effect of chloride additives on the XRD pattern of the CsFAPI familly films. The films are produced by spin-coating, (a-c) before annealing, (d-f) after 4 s- 5 s of annealing and (g-i) after full annealing. XRD of CsFAPI/AKC layer after 40s of spin-coating without (j) and with (k) chlorobenzene drop. Source data are provided as a Source Data file.

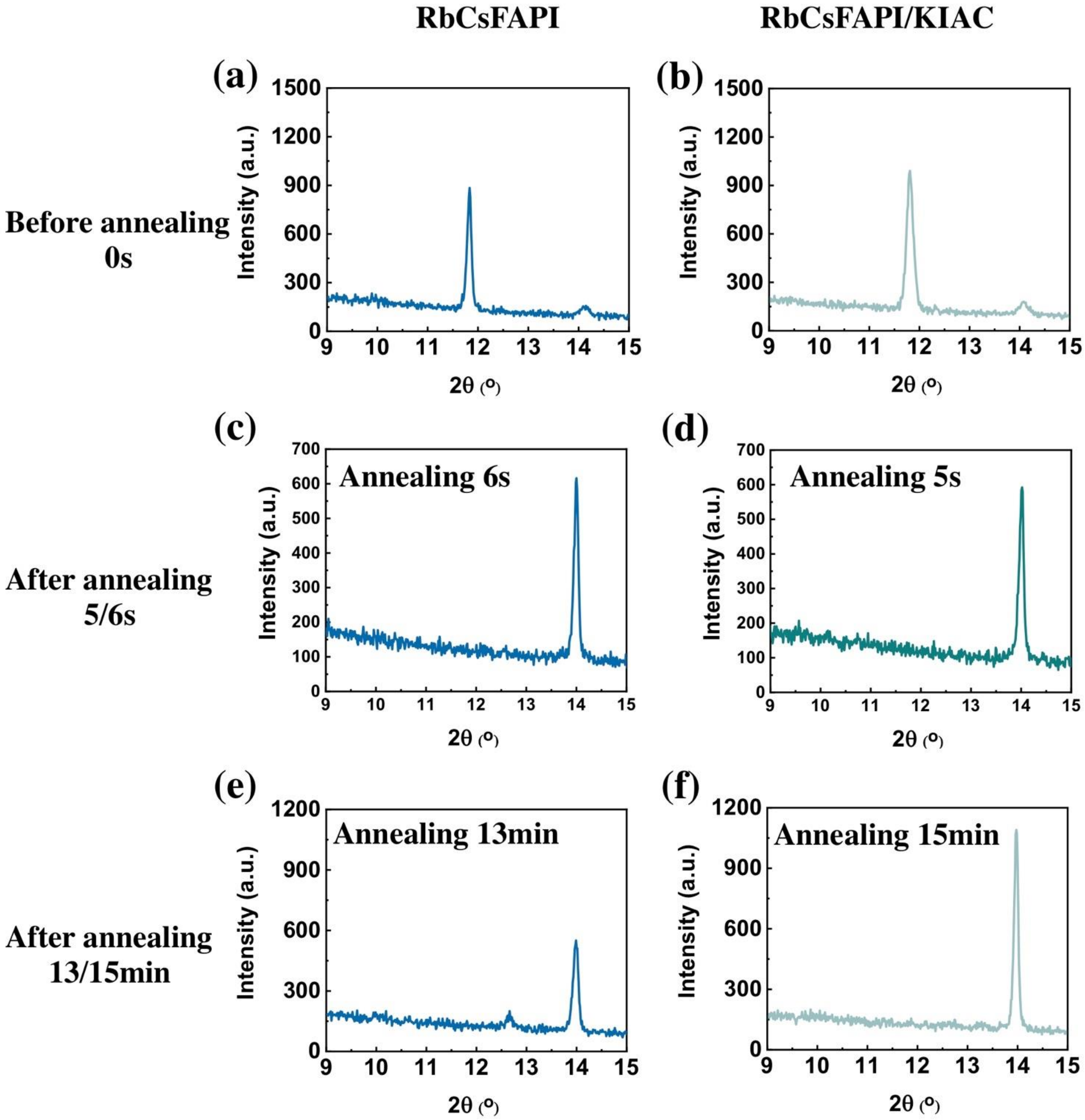

Supplementary Fig. 8: Effect of additives on the XRD pattern of the RbCsFAPI familly films. They are produced by spin-coating, before annealing (a-b), after 5 s-6 s of annealing (c-d) and after (e-f) full annealing. Source data are provided as a Source Data file.

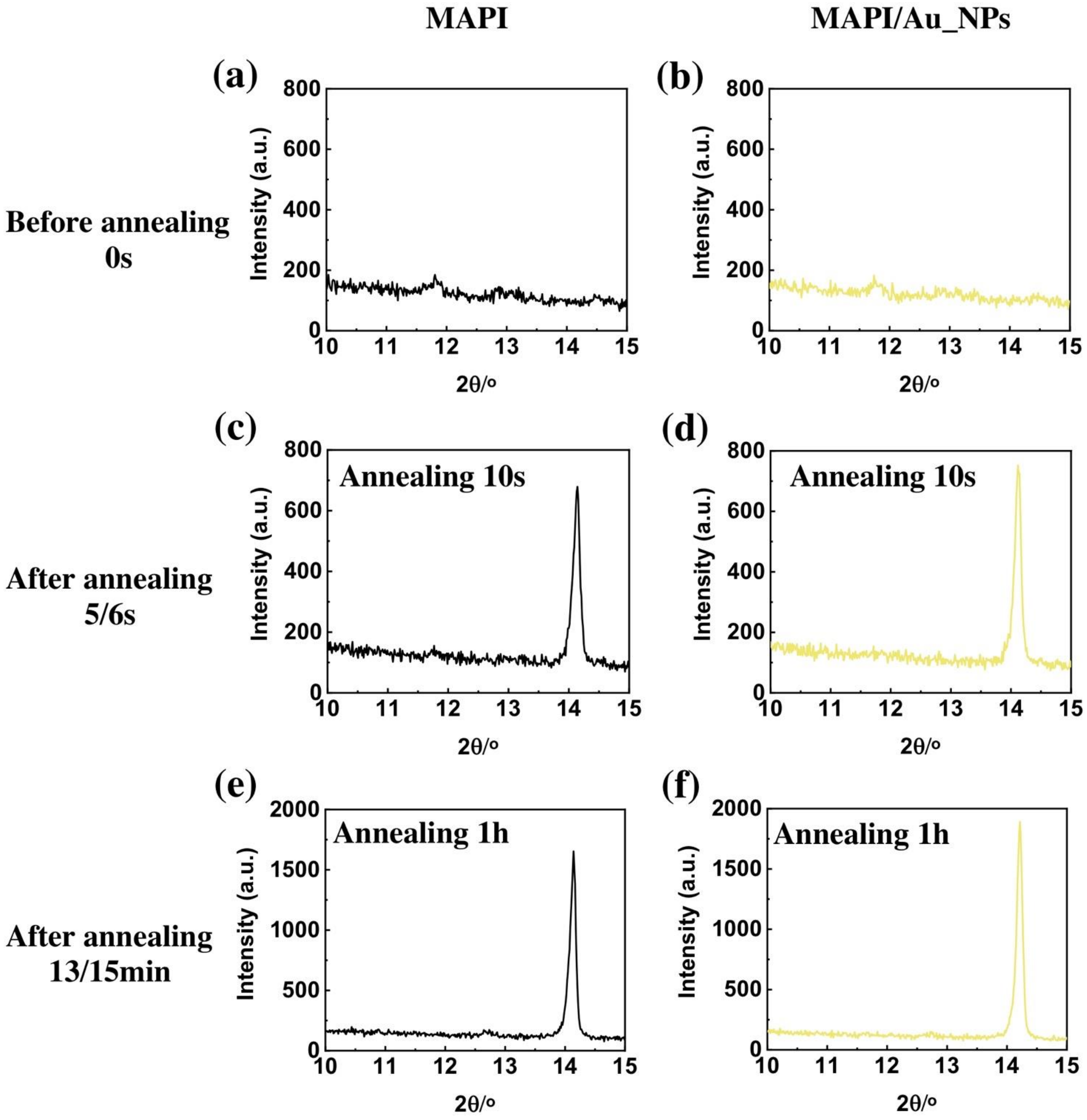

Supplementary Fig. 9: Effect of Au_NPs on the XRD pattern of the MAPI films. They are produced by spin-coating, before annealing (a-b), after 10 s of annealing (c-d) and after (e-f) full annealing. Source data are provided as a Source Data file.

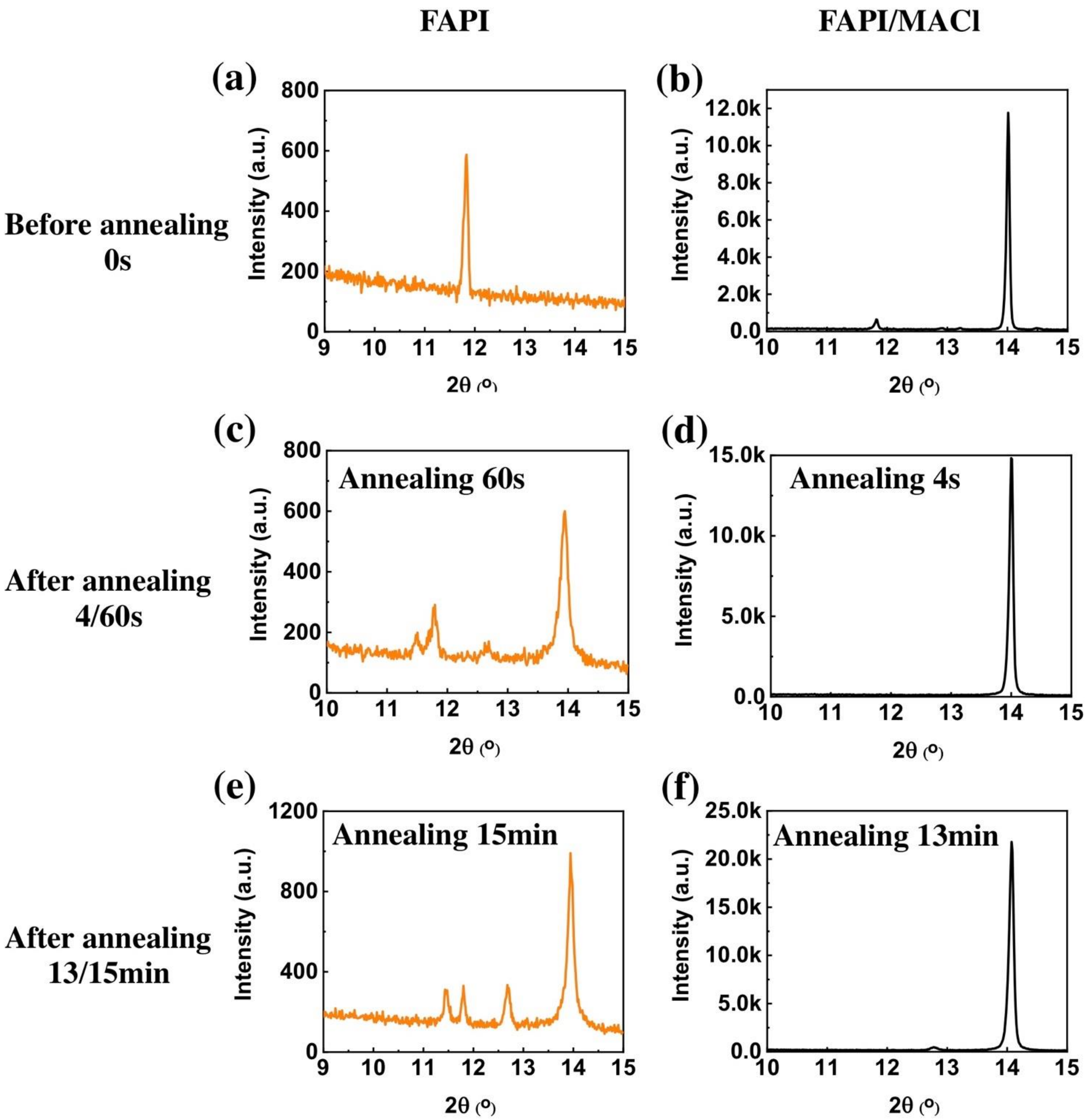

Supplementary Fig. 10: Effect of MACl on the XRD pattern of the FAPI films. They produced by spin-coating, before annealing (a-b), after 60 s- 4 s of annealing (c-d) and after (e-f) full annealing. Source data are provided as a Source Data file.

Supplementary Table 2: Time of color change and total annealing time of the different investigated perovskites.

STAGE	Time of Annealing	CsFAPI	CsFAPI /KC	CsFAPI /AKC	RbCsFAPI	RbCsFAPI /KIAC	MAPI	MAPI /Au_NPs	FAPI	FAPI /MACl
Stage-1	Before annealing	0s	0s	0s	0s	0s	0s	0s	0s	0s
	Color changes	5s	4s	5s	6s	5s	10s	10s	1min	4s
Stage-2	End of annealing	13min	13min	15min	13min	15min	1h	1h	15min	15min

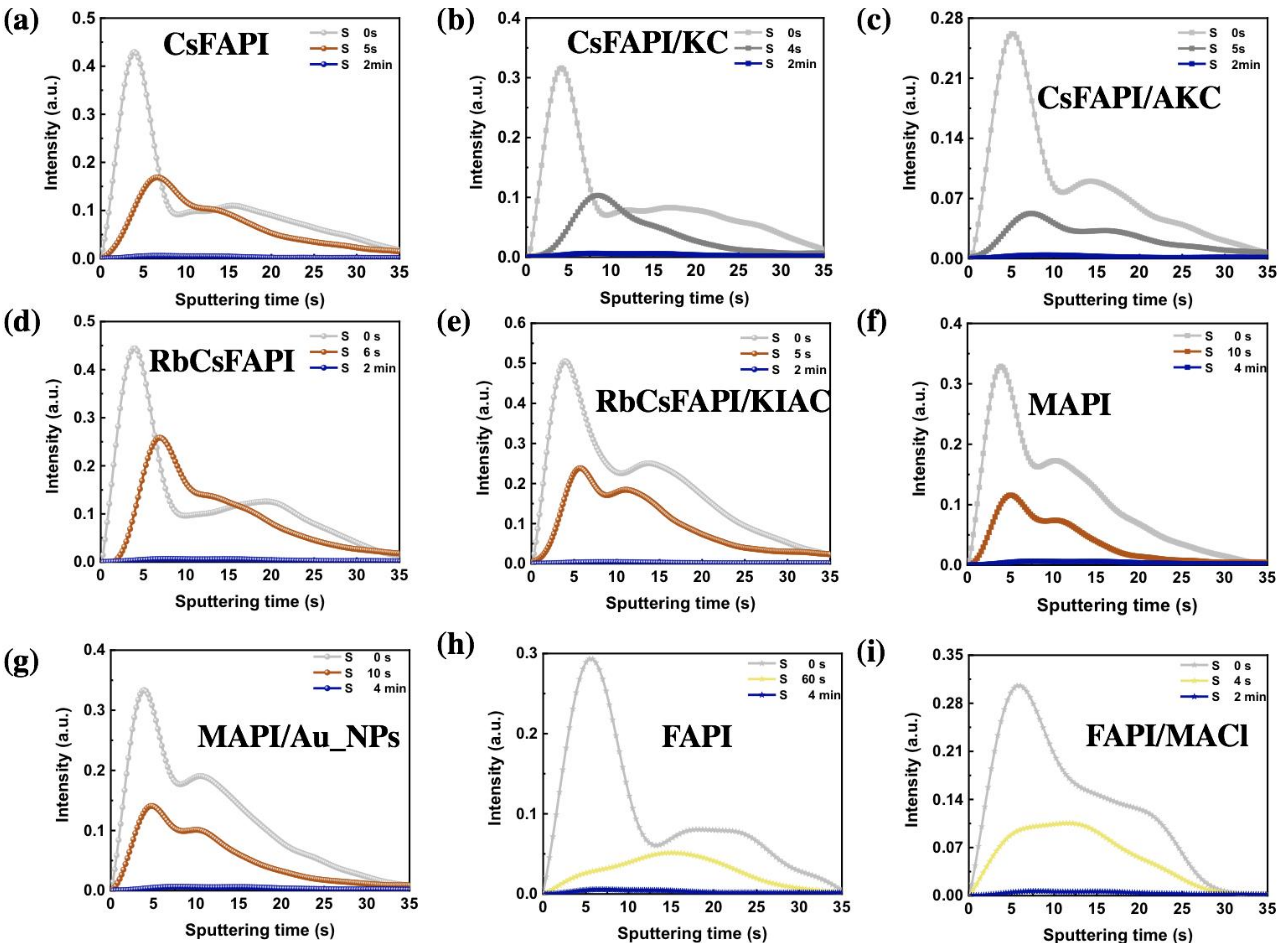

Supplementary Fig. 11: GD-OES S profile. The various films were annealed between 0 s and 2 min (or 4 min). Source data are provided as a Source Data file.

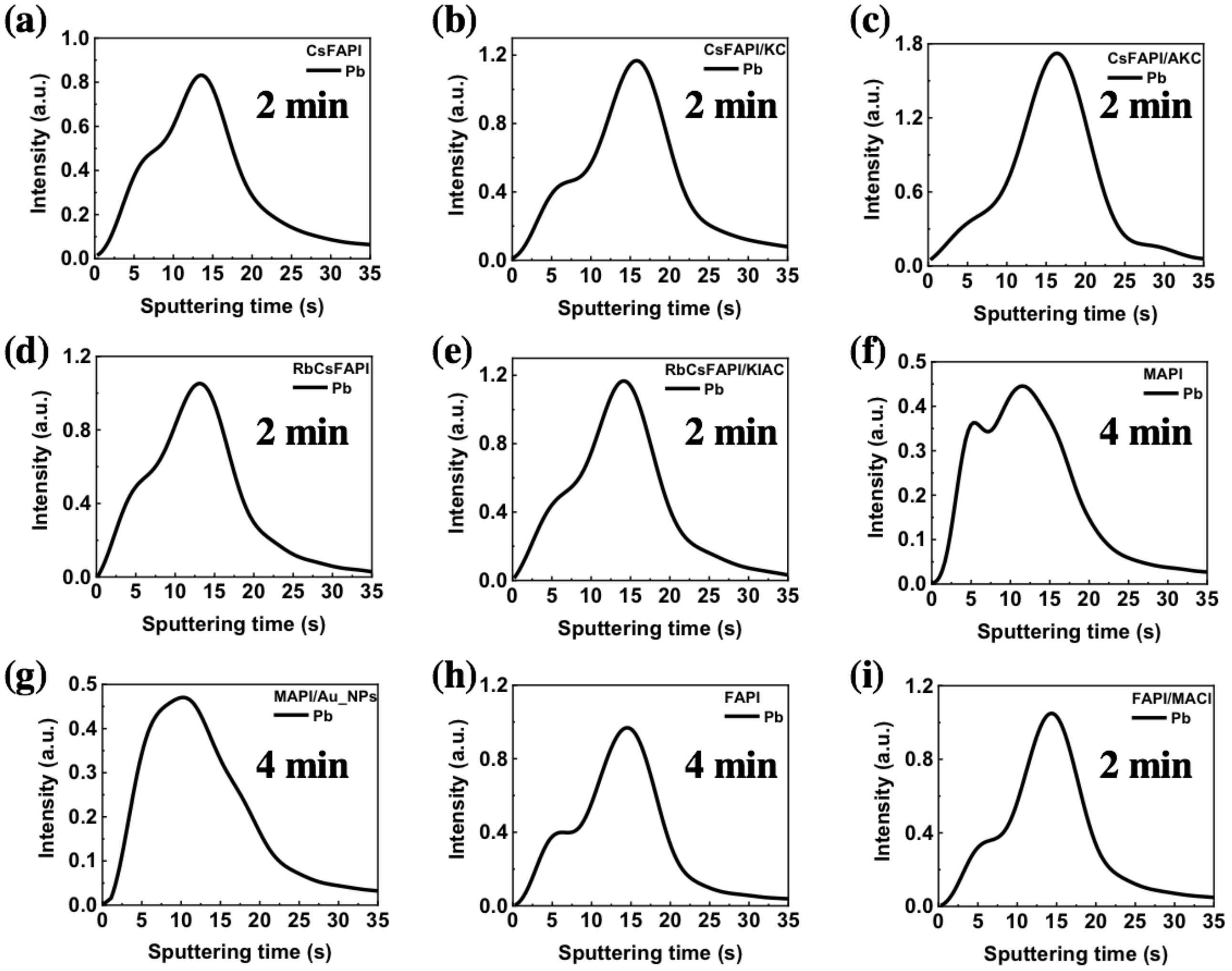

Supplementary Fig. 12: GD-OES Pb profiles. The films were annealed between 0s and 2 min (or 4 min). Source data are provided as a Source Data file.

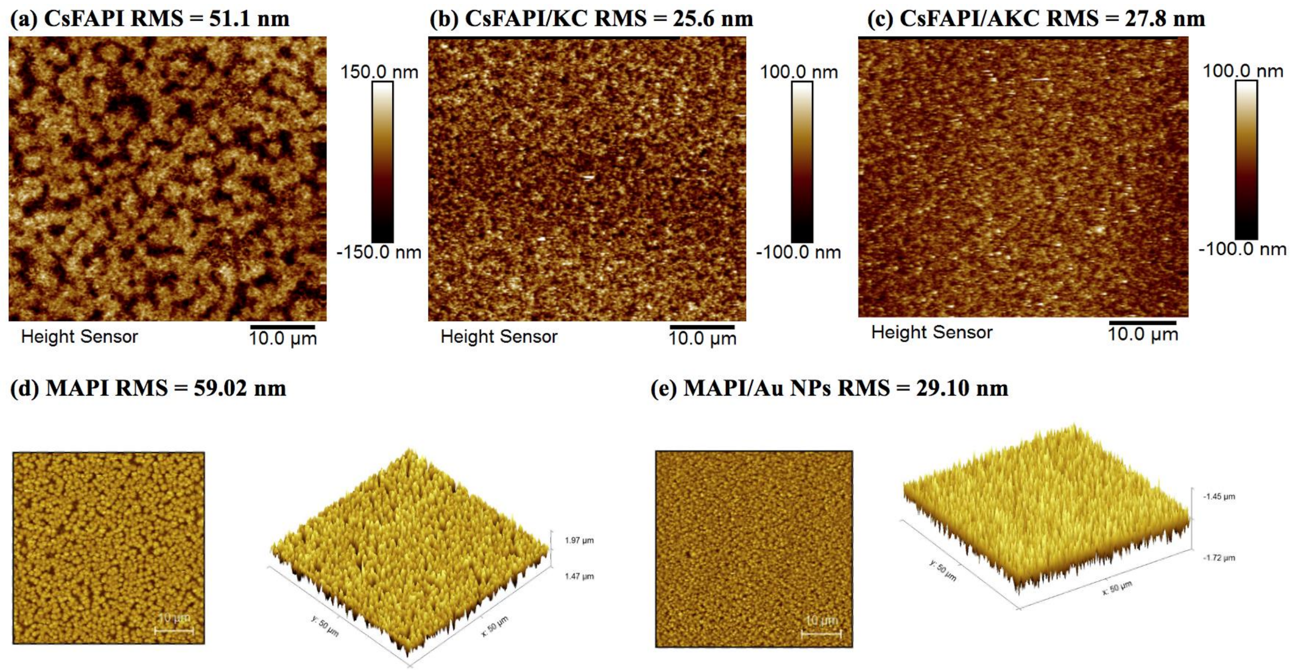

Supplementary Fig. 13: AFM measurement of surface roughness. Perovskite samples prepared without and with growth additives.

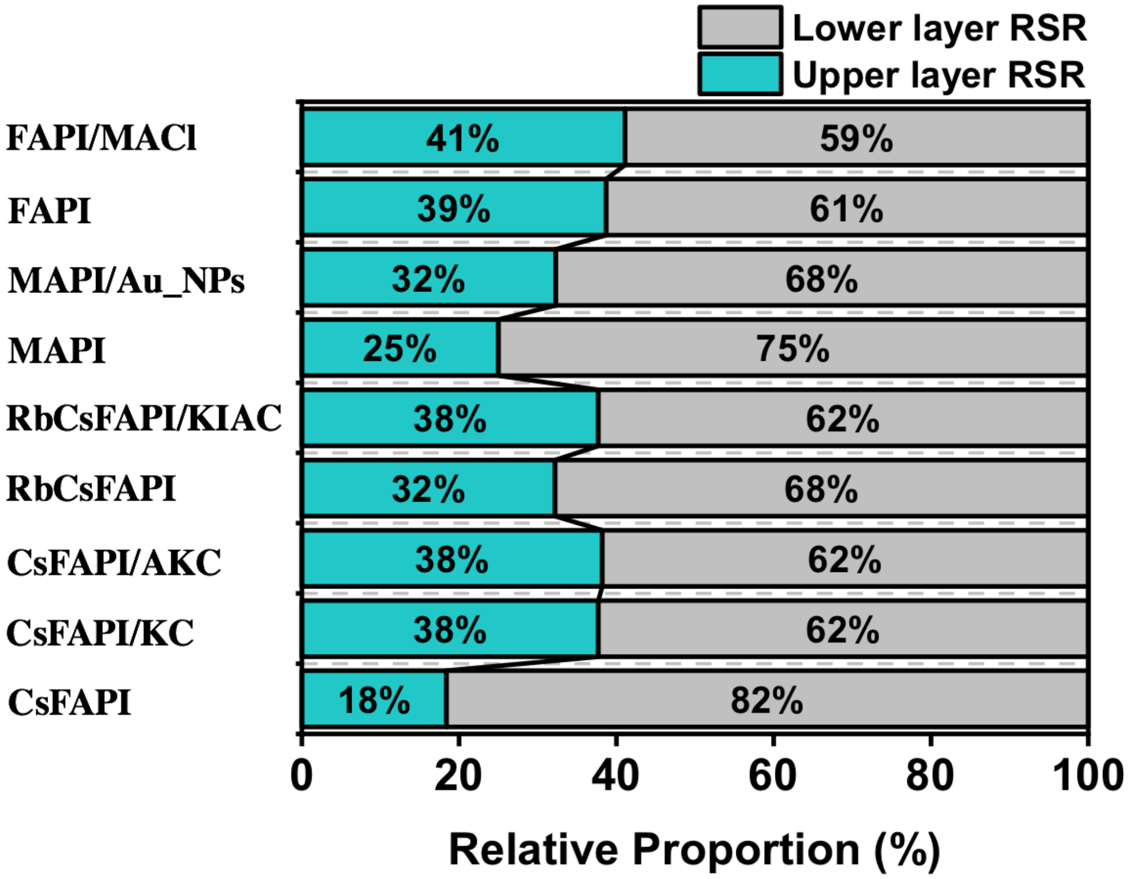

Supplementary Fig. 14: Relative proportion of upper layer and lower layer at late annealing time. Upper layer RSR is in blue and Lower layer RSR is in grey. Source data are provided as a Source Data file.

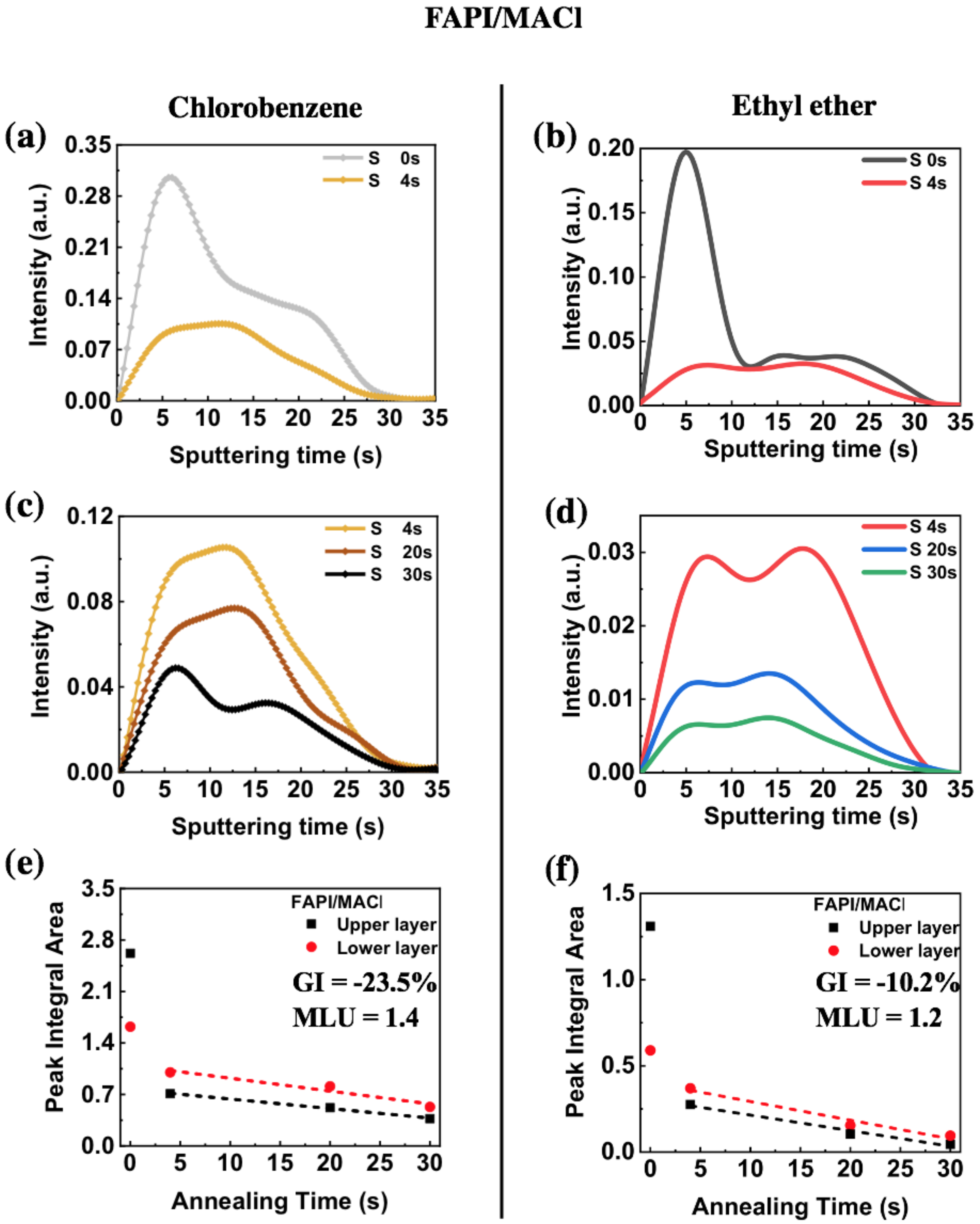

Supplementary Fig. 15: Effect of antisolvent on the S element profile upon annealing. (a-d) Comparison of chlorobenzene and diethyl ether (DEE) antisolvent effects on the GD-OES profiles of S element in the layer at increasing thermal annealing times. Peak integral area for the upper and lower layers for CB (e) and DEE (f) antisolvents. Source data are provided as a Source Data file.

Supplementary Reference

[1] Zheng, D. et al. What are Methylammonium and Solvent Fates upon Halide Perovskite Thin-Film Preparation and Thermal Aging? *Adv. Mater. Interfaces* 2201436 (2022).
